# Future-proofing and maximizing the utility of metadata: The PHA4GE SARS-CoV-2 contextual data specification package

**DOI:** 10.1093/gigascience/giac003

**Published:** 2022-02-16

**Authors:** Emma J Griffiths, Ruth E Timme, Catarina Inês Mendes, Andrew J Page, Nabil-Fareed Alikhan, Dan Fornika, Finlay Maguire, Josefina Campos, Daniel Park, Idowu B Olawoye, Paul E Oluniyi, Dominique Anderson, Alan Christoffels, Anders Gonçalves da Silva, Rhiannon Cameron, Damion Dooley, Lee S Katz, Allison Black, Ilene Karsch-Mizrachi, Tanya Barrett, Anjanette Johnston, Thomas R Connor, Samuel M Nicholls, Adam A Witney, Gregory H Tyson, Simon H Tausch, Amogelang R Raphenya, Brian Alcock, David M Aanensen, Emma Hodcroft, William W L Hsiao, Ana Tereza R Vasconcelos, Duncan R MacCannell

**Affiliations:** Faculty of Health Sciences, Simon Fraser University, Burnaby V5A 1S6, BC, Canada; Center for Food Safety and Applied Nutrition, U.S. Food and Drug Administration, College Park, MD 20740, USA; Instituto de Microbiologia, Instituto de Medicina Molecular, Faculdade de Medicina, Universidade de Lisboa, Lisboa 1649-028, Portugal; Microbes in the Food Chain, Quadram Institute Bioscience, Norwich, Norfolk NR4 7UQ, UK; Microbes in the Food Chain, Quadram Institute Bioscience, Norwich, Norfolk NR4 7UQ, UK; BC Centre for Disease Control Public Health Laboratory, Vancouver, BC V5Z 4R4, Canada; Faculty of Computer Science, Dalhousie University, Halifax, NS B3H 1W5, Canada; INEI-ANLIS “Dr Carlos G. Malbrán,” Buenos Aires C1282AFF, Argentina; Infectious Disease and Microbiome Program, The Broad Institute of MIT and Harvard, Cambridge, MA 02142, USA; African Center of Excellence for Genomics of Infectious Diseases (ACEGID), Redeemer's University, Ede, Osun State 232103, Nigeria; Department of Biological Sciences, College of Natural Sciences, Redeemer's University, Ede, Osun State 232103, Nigeria; African Center of Excellence for Genomics of Infectious Diseases (ACEGID), Redeemer's University, Ede, Osun State 232103, Nigeria; Department of Biological Sciences, College of Natural Sciences, Redeemer's University, Ede, Osun State 232103, Nigeria; South African Medical Research Council Bioinformatics Unit, South African National Bioinformatics Institute, University of the Western Cape, Bellville 7530, South Africa; South African Medical Research Council Bioinformatics Unit, South African National Bioinformatics Institute, University of the Western Cape, Bellville 7530, South Africa; Microbiological Diagnostic Unit Public Health Laboratory, The Peter Doherty Institute for Infection and Immunity, The University of Melbourne, Melbourne, VIC 3000, Australia; Faculty of Health Sciences, Simon Fraser University, Burnaby V5A 1S6, BC, Canada; Faculty of Health Sciences, Simon Fraser University, Burnaby V5A 1S6, BC, Canada; Center for Food Safety, University of Georgia, Atlanta, GA 30333, USA; Office of Advanced Molecular Detection, National Center for Emerging and Zoonotic Infectious Diseases, Centers for Disease Control and Prevention, GA 30333, USA; Department of Epidemiology, University of Washington, WA 98109, USA; National Center for Biotechnology Information, National Library of Medicine, National Institutes of Health, Bethesda, MD 20894, USA; National Center for Biotechnology Information, National Library of Medicine, National Institutes of Health, Bethesda, MD 20894, USA; National Center for Biotechnology Information, National Library of Medicine, National Institutes of Health, Bethesda, MD 20894, USA; Organisms and Environment Division, School of Biosciences, Cardiff University, Cardiff CF10 3AX, UK; Public Health Wales, University Hospital of Wales, Cardiff CF14 4XW, UK; University of Birmingham, Birmingham B17 2TT, UK; Institute for Infection and Immunity, St George's, University of London, London SW17 0RE, UK; Center for Veterinary Medicine, U.S. Food and Drug Administration, Laurel, MD 20708, USA; Department of Biological Safety, German Federal Institute for Risk Assessment, Berlin 12277, Germany; Department of Biochemistry and Biomedical Sciences and the Michael G. DeGroote Institute for Infectious Disease Research, McMaster University, Hamilton, ON L8S 4L8, Canada; Department of Biochemistry and Biomedical Sciences and the Michael G. DeGroote Institute for Infectious Disease Research, McMaster University, Hamilton, ON L8S 4L8, Canada; Centre for Genomic Pathogen Surveillance, Wellcome Genome Campus, Cambridge CB10 1SA, UK; The Big Data Institute, Li Ka Shing Centre for Health Information and Discovery, Nuffield Department of Medicine, University of Oxford, Oxford OX3 7LF, UK; Biozentrum, University of Basel, Basel 3012, Switzerland; Swiss Institute of Bioinformatics, Lausanne, Switzerland; Faculty of Health Sciences, Simon Fraser University, Burnaby V5A 1S6, BC, Canada; BC Centre for Disease Control Public Health Laboratory, Vancouver, BC V5Z 4R4, Canada; Department of Pathology and Laboratory Medicine, University of British Columbia, Vancouver, BC V6T 1Z7 V6T 1Z7, Canada; Bioinformatics Laboratory National Laboratory of Scientific Computation LNCC/MCTI, Petrópolis 25651-075, Brazil; Office of Advanced Molecular Detection, National Center for Emerging and Zoonotic Infectious Diseases, Centers for Disease Control and Prevention, GA 30333, USA

**Keywords:** genomics, metadata, SARS-CoV-2, bioinformatics, data standards

## Abstract

**Background:**

The Public Health Alliance for Genomic Epidemiology (PHA4GE) (https://pha4ge.org) is a global coalition that is actively working to establish consensus standards, document and share best practices, improve the availability of critical bioinformatics tools and resources, and advocate for greater openness, interoperability, accessibility, and reproducibility in public health microbial bioinformatics. In the face of the current pandemic, PHA4GE has identified a need for a fit-for-purpose, open-source SARS-CoV-2 contextual data standard.

**Results:**

As such, we have developed a SARS-CoV-2 contextual data specification package based on harmonizable, publicly available community standards. The specification can be implemented via a collection template, as well as an array of protocols and tools to support both the harmonization and submission of sequence data and contextual information to public biorepositories.

**Conclusions:**

Well-structured, rich contextual data add value, promote reuse, and enable aggregation and integration of disparate datasets. Adoption of the proposed standard and practices will better enable interoperability between datasets and systems, improve the consistency and utility of generated data, and ultimately facilitate novel insights and discoveries in SARS-CoV-2 and COVID-19. The package is now supported by the NCBI’s BioSample database.

## Findings

### The importance of contextual data for interpreting SARS-CoV-2 sequences

First identified in late 2019 in Wuhan, China, the SARS-CoV-2 virus has now spread to virtually every country and territory in the world, resulting in millions of confirmed cases, and deaths, globally [1, 2]. Understanding, monitoring, and preventing transmission, as well as the development of vaccines and effective therapeutic options, have been primary goals of the public health response to SARS-CoV-2.

Tracking the spread and evolution of the virus at global, national, and local scales has been aided by the analysis of viral genome sequence data alongside SARS-CoV-2 epidemiology. Large-scale sequencing efforts are often formalized as consortia across the world, including the COG-UK in the UK [3], SPHERES in the USA [4], CanCOGeN in Canada [5], the Latin American Genomics SARS-CoV-2 Network [6, 7], 2019nCoVR in China [8], the South Africa NGS Genomic Surveillance Network [9], AusTrakka in Australia and New Zealand [10], and INSACOG in India [11]. In addition to these initiatives, many agencies, universities, and hospital laboratories around the world are also sequencing and sharing sequence data at an unprecedented pace. Deposition of these sequences into public repositories such as the Global Initiative on Sharing All Influenza Data (GISAID) and the International Nucleotide Sequence Database Collaboration (INSDC) has enabled rapid global sharing of data [12, 13]. At the time of writing, 174 countries had undertaken open sequencing initiatives (GISAID accessed 2021–06-23) depositing 2,057,675 sequences, which are being reused and analysed on a massive scale. The open data sharing paradigm has had tremendous success in the genomic epidemiology of foodborne pathogens [14, 15] and has the potential to reveal a deeper understanding of SARS-CoV-2 origin, pathogenicity, and basic biological characteristics when submissions from environmental samples and wild hosts are included alongside human clinical samples [16].

SARS-CoV-2 sequencing, analysis, and open sharing have played a crucial role in a number of developments during the pandemic, such as dispelling misinformation about the origins of the virus [17], the identification and surveillance of variants of concern [18, 19], the improvement of diagnostic performance and rapid testing [20–22], and the development of vaccines, which are currently being distributed in the largest global vaccination program the world has ever seen [23]. Viral genomic sequences are also being used to understand transmission and reinfection events [24], as well to monitor the prevalence and diversity of lineages during different exposure events and in different settings, e.g., animal reservoirs [25], long-term care facilities [26–28], healthcare and other work sites [29–33], and conferences and other public gatherings [34], as well as before and after public health responses (e.g., border controls and travel restrictions, lockdowns and quarantines, vaccination), through successive waves of infections [35–46]. However, it is critical to note that public health sequence data are of limited value without accompanying contextual metadata.

Contextual data consist of sample metadata (e.g., collection date, sample type, geographical location of sample collection), as well as laboratory (e.g., date and location testing, cycle threshold [CT] values), clinical outcomes (e.g., hospitalization, death, recovery), epidemiological (e.g., age, sex, exposures, vaccination status), and methods (e.g., sampling, sequencing, bioinformatics) data that enable the interpretation of sequence data. High-quality contextual data are also crucial for quality control. For example, detecting systematic batch effect errors related to certain sequencing centres and methods can help evaluate which variants represent real, circulating viruses, as opposed to artefacts of sample handling or sequencing that may arise owing to different aspects of experimental design, laboratory procedures, bioinformatics processing, and applied quality control thresholds [47–49].

Good data stewardship practices are critical not only for auditability and reproducibility but for posterity—documenting critical information about samples, methods, risk factors and outcomes, and so forth can help future-proof information used to build a roadmap for dealing with future public health crises. Contextual data, however, are often collected on a project-specific basis according to local needs and reporting requirements, which results in the collection of different data types at different levels of granularity, with different meanings and implicit bias of variables and attributes. Furthermore, the information is often collected as free text or, if structured, according to organization or initiative-specific data dictionaries, using different fields, terms, formats, abbreviations, and jargon.

The variability in the way information is encoded in private databases tends to propagate to public repositories, which makes the information more difficult to interpret and to use. There are different existing standards that can be used to structure contextual data, like minimum information checklists (MIxS [50], MIGS [51], the NIAID/BRC Project, and Sample Application Standard [52]) and various interoperable ontologies (OBO Foundry [53]), which make information easier to aggregate and reuse for different types of analyses. However, these attribute packages and metadata standards developed by different organizations are usually scoped to cover as many use cases and pathogens as possible and, as such, can include fields of information not applicable to SARS-CoV-2, or that may be subject to privacy concerns, or exclude fields commonly used in public health surveillance and investigations. Because different types of contextual data are subject to different ethical, practical, and privacy concerns, not all components of existing standards are immediately or widely collectable and shareable. As a result, the range of generic metadata standards being applied to SARS-CoV-2 data presents challenges for data harmonization [54] and analysis critical for fighting the disease and ending the pandemic.

In light of these challenges, PHA4GE has identified a need for a fit-for-purpose, open-source SARS-CoV-2 contextual data specification that can be used to consistently structure information as part of good data management practices and for data sharing with trusted partners and/or public repositories. The specification was developed by consensus among domain experts, and incorporates existing community standards with an emphasis on SARS-CoV-2 public health needs and ensuring privacy while maximizing information content and interoperability across datasets and databases to better enable analyses to fight COVID-19. The specification package also contains a number of accompanying materials such as standard operating procedures, tools, a reference guide, and repository submission protocols (protocols.io) to help put the standard into practice.

## SARS-CoV-2 Contextual Data Specification: The Framework

**Figure 1:**
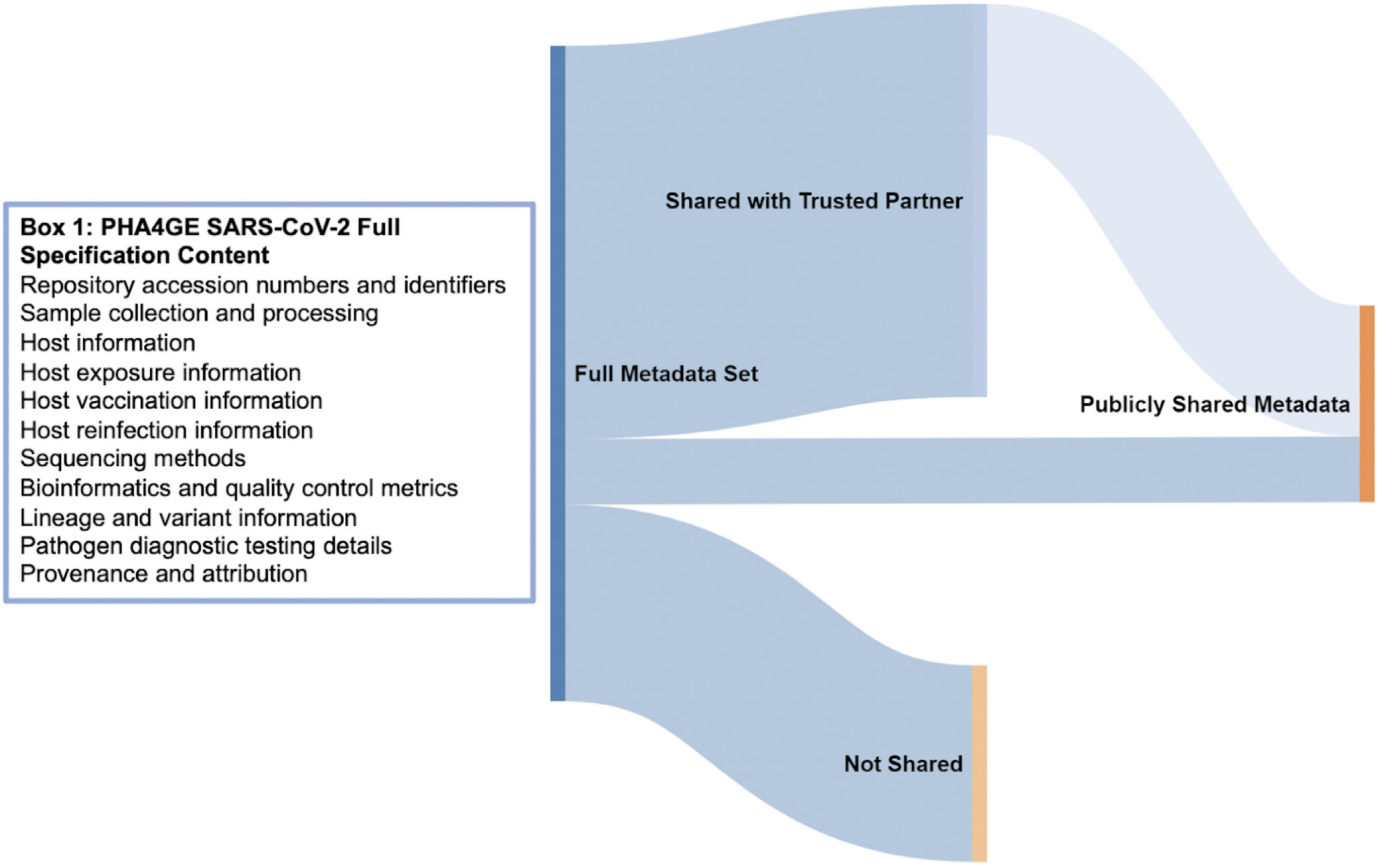
: Contextual data flow. Contextual data can be captured and structured using the PHA4GE specification so that they can be more easily harmonized across different data sources and providers. Different subsets of the harmonized data can be (i) shared with public repositories, e.g., GISAID and INSDC; (ii) shared with trusted partners, e.g., national sequencing consortia, public health partners; and (iii) kept private and retained locally with the potential for sharing in the future for particular surveillance or research activities. While fields have been colour-coded in the template to indicate whether they are considered “required,” “strongly recommended,” or “optional,” how the specification is implemented and whether any of the data are shared is ultimately at the discretion of the user. Box 1 describes the information types covered in the full specification.

The purpose of the PHA4GE SARS-CoV-2 specification is to provide a mechanism for consistent structure, collection, and formatting of fields and values containing SARS-CoV-2 contextual data pertaining to clinical, animal, and environmental samples. We emphasize that the purpose of this specification is not to force data sharing but rather to provide a framework to structure data consistently across disparate laboratory and epidemiological databases so that they can be harmonized for different uses (Fig. 1). Data sharing is just one use case and can involve sharing between divisions within a single agency, sharing between partners based on memorandums of understanding, or submission to public repositories.

The PHA4GE SARS-CoV-2 contextual data specification was created through broad consultation with representatives from public health laboratories, research institutes, and universities in 11 countries (Argentina, Australia, Brazil, Canada, Germany, Nigeria, Portugal, South Africa, Switzerland, the United Kingdom, the United States of America) who are involved with SARS-CoV-2 genome sequencing and analysis efforts at various scales. Based on this consultation and consensus, the specification contains different fields covering a wide array of data types described in Box 1 (Fig. 1). The specification attempts to harmonize different data standards (e.g., INSDC, GISAID, MIxS, MIGS, Sample Application Standard) by reusing fields or mapping to fields, as much as possible. Because PHA4GE embraces FAIR data stewardship principles (Findability, Accessibility, Interoperability, and Reuse of digital assets), we strived to implement FAIR principles in the design and implementation of the specification for data management and data sharing. At their core, these principles emphasize machine-actionability and consistency of data and are critical for dealing with the volume and complexity of genomic sequence and contextual data. Principles of FAIR data stewardship that have been implemented include improving machine-actionability of data by using a formal, accessible, shared, and broadly applicable language for knowledge representation, reusing existing standards and ontology-based vocabulary to increase interoperability, providing a data use license, capturing data provenance, and making all resources open, free, and widely accessible.

The versioned specification is available as a contextual data collection template (.xlsx) and in machine-amenable JSON format from GitHub (version 3.0.0) [55]. The collection template also offers standardized terms for a number of fields in the form of pick lists. The fields are colour-coded to indicate required (yellow), strongly recommended (purple), or optional status (white). Fields useful for surveillance were prioritized as “required”. Formats for data elements like dates are also prescribed according to international standards (e.g., dates should be formatted according to ISO 8601).

The template is also supported by several materials such as term and field-level Reference Guides (available as tabs in the collection template Excel workbook), which provide definitions, data entry guidance, and examples of usage [55]. The field-level Reference Guide also provides mapping of PHA4GE fields to existing contextual data standards, highlighting public health and SARS-CoV-2–specific fields that were missing, as well as fields in those other standards that were considered out of scope.

The Open Biological and Biomedical Ontology (OBO) Foundry is a community of researchers who use a prescribed set of principles and practices to develop a wide range of interoperable ontologies focused on the life sciences [56]. Fields and terms in the specification have been mapped to existing OBO Foundry ontology terms, and where required, new ontology terms have been developed and are being made available in different application and domain-specific ontologies within The Foundry (see Table 1 for a list of source ontologies). As of version 3.0.0 and beyond, terms in pick lists provided in the collection template are presented with corresponding ontology identifiers in the format “Label [ontology ID]”, e.g., Blood [UBERON:0 000 178]. Axioms and additional cross references to ontologies and existing standards are actively being developed in collaboration with community developers. We anticipate that our contributions to these freely available, open-source resources will be of use to the COVID-19 research community.

**Table 1:** : Ontologies implemented in the PHA4GE SARS-CoV-2 specification

Ontology^1^	Link
BRENDA Tissue Ontology (BTO)	https://obofoundry.org/ontology/bto.html
Cell Line Ontology (CLO)	https://obofoundry.org/ontology/clo.html
Environmental conditions, treatments and exposures ontology (ECTO)	https://obofoundry.org/ontology/ecto.html
Environment Ontology (ENVO)	https://obofoundry.org/ontology/envo.html
Food Ontology (FoodOn)	https://obofoundry.org/ontology/foodon.html
Gazetteer Ontology (GAZ)	https://obofoundry.org/ontology/gaz.html
Gender, Sex, and Sexual Orientation Ontology (GSSO)	https://obofoundry.org/ontology/gsso.html
Genomic Epidemiology Ontology (GenEpiO)	https://obofoundry.org/ontology/genepio.html
Genomics Cohorts Knowledge Ontology (GECKO)	https://obofoundry.org/ontology/gecko.html
Human Disease Ontology (DOID)	https://obofoundry.org/ontology/doid.html
Human Phenotype Ontology (HP)	https://obofoundry.org/ontology/hp.html
Mammalian Phenotype Ontology (MP)	https://obofoundry.org/ontology/mp.html
Measurement Method Ontology (MMO)	https://obofoundry.org/ontology/mmo.html
Mondo Disease Ontology (MONDO)	https://obofoundry.org/ontology/mondo.html
Mouse Pathology Ontology (MPATH)	https://obofoundry.org/ontology/mpath.html
National Cancer Institute Thesaurus (NCIT)	https://obofoundry.org/ontology/ncit.html
NCBI Taxonomy Ontology (NCBITaxon)	https://obofoundry.org/ontology/ncbitaxon.html
Neuro Behaviour Ontology (NBO)	https://obofoundry.org/ontology/nbo.html
Ontology for Biomedical Investigations (OBI)	https://obofoundry.org/ontology/obi.html
Ontology of Medically Related Social Entities (OMRSE)	https://obofoundry.org/ontology/omrse.html
Population and Community Ontology (PCO)	https://obofoundry.org/ontology/pco.html
UBERON Multi-species Anatomy Ontology (UBERON)	https://obofoundry.org/ontology/uberon.html
Unit Ontology (UO)	https://obofoundry.org/ontology/uo.html
Vaccine Ontology (VO)	https://obofoundry.org/ontology/vo.html

1 Vocabulary for fields and terms in the specification have been sourced or mapped to OBO Foundry domain and application ontologies, which are highlighted in this list. New fields and terms for which there were no existing equivalents have been developed and submitted to these ontologies, expanding these community resources.

Protocols have also been created and are openly available on protocols.io [57], including a curation Standard Operating Procedure (SOP) containing instructions for using the collection template, as well as guidance for a number of privacy and practical concerns. A series of versioned SARS-CoV-2 sequence and contextual data submission protocols and accompanying instructional videos for how to prepare submissions and navigate through the various submission portals for GISAID, NCBI, and EMBL-EBI are also provided.

A mapping file indicating which PHA4GE fields correspond to contextual data elements recommended by the World Health Organization has been provided to help data providers comply with international guidance [58]. This mapping file also includes tabs indicating which PHA4GE fields correspond to those found in different repository submission forms to facilitate data transformations for submissions. Such transformations can be automated using a contextual data harmonization application called the DataHarmonizer [59]. PHA4GE has worked with the developers of the DataHarmonizer to offer the PHA4GE standard as a template in the tool (I. Gill et al., in preparation). Users can standardize and validate entered data and export it as GISAID and NCBI-ready submission forms (BioSample, SRA, GenBank, and GenBank source modifier forms). It should be noted that other excellent contextual data transformation tools have been developed by the community, such as METAGENOTE, multiSub, and a GISAID-to-ENA conversion script [60–62].

The different specification package materials are outlined in Table 2.

**Table 2:** : Resources that form the PHA4GE SARS-CoV-2 contextual data specification package [55]

Resource^1^	Description	Link
Collection template and controlled vocabulary pick lists	Spreadsheet-based collection form containing different fields (identifiers and accessions, sample collection and processing, host information, host exposure, vaccination and reinfection information, lineage and variant information, sequencing, bioinformatics and quality control metrics, diagnostic testing information, author acknowledgements). Fields are colour-coded to indicate required, recommended, or optional status. Many fields offer pick lists of controlled vocabulary. Vocabulary lists are also available in a separate tab	https://github.com/pha4ge/SARS-CoV-2-Contextual-Data-Specification/raw/master/PHA4GE%20SARS-CoV-2%20Contextual%20Data%20Template.xls
Reference guides	Field and term definitions, guidance, and examples are provided as separate tabs in the collection template .xlsx file (see Term Reference Guide and Field Reference Guide)	https://github.com/pha4ge/SARS-CoV-2-Contextual-Data-Specification/raw/master/PHA4GE%20SARS-CoV-2%20Contextual%20Data%20Template.xlsx
Curation protocol on protocols.io	Step-by-step instructions for using the collection template are provided in an SOP. Ethical, practical, and privacy considerations are also discussed. Examples and instructions for structuring sample descriptions as well as sourcing additional standardized terms (outside those provided in pick lists) are also discussed	dx.doi.org/10.17504/protocols.io.btpznmp6
Mapping file of PHA4GE fields to metadata standards	PHA4GE fields are mapped to existing metadata standards such as the Sample Application Standard, MIxS 5.0, and the MIGS Virus Host-associated attribute package. Mappings are available in the Reference guide tab. Mappings highlight which fields of these standards are considered useful for SARS-CoV-2 public health surveillance and investigations, and which fields are considered out of scope	https://github.com/pha4ge/SARS-CoV-2-Contextual-Data-Specification/raw/master/PHA4GE%20SARS-CoV-2%20Contextual%20Data%20Template.xlsx
Mapping of PHA4GE fields to WHO metadata recommendations	PHA4GE fields are mapped to corresponding contextual data elements recommended by the World Health Organization	https://github.com/pha4ge/SARS-CoV-2-Contextual-Data-Specification/blob/master/PHA4GE%20to%20WHO%20and%20Sequence%20Repository%20Field%20Mappings.xlsx
Mapping file of PHA4GE fields to EMBL-EBI, NCBI, and GISAID submission requirements	Many PHA4GE fields have been sourced from public repository submission requirements. The different repositories have different requirements and field names. Repository submission fields have been mapped to PHA4GE fields to demonstrate equivalencies and divergences.	https://github.com/pha4ge/SARS-CoV-2-Contextual-Data-Specification/blob/master/PHA4GE%20to%20WHO%20and%20Sequence%20Repository%20Field%20Mappings.xlsx
Data submission protocol (NCBI) on protocols.io	The SARS-CoV-2 submission protocol for NCBI provides step-by-step instructions and recommendations aimed at improving interoperability and consistency of submitted data	dx.doi.org/10.17504/protocols.io.bui7nuhn
Data submission protocol (EMBL-EBI) on protocols.io	The SARS-CoV-2 submission protocol for ENA provides step-by-step instructions and recommendations aimed at improving interoperability and consistency of submitted data	dx.doi.org/10.17504/protocols.io.buqnnvve
Data submission protocol (GISAID) on protocols.io	The SARS-CoV-2 submission protocol for GISAID provides step-by-step instructions and recommendations aimed at improving interoperability and consistency of submitted data	dx.doi.org/10.17504/protocols.io.bumknu4w
JSON structure of PHA4GE specification	A JSON structure of the PHA4GE specification has been provided for easier integration into software applications	https://raw.githubusercontent.com/pha4ge/SARS-CoV-2-Contextual-Data-Specification/master/PHA4GE_SARS-CoV-2_Contextual_Data_Schema.json
PHA4GE template in the DataHarmonizer	Javascript application enabling standardized data entry, validation, and export of contextual data as submission-ready forms for GISAID and NCBI. The SOP for using the software can be found at https://github.com/Public-Health-Bioinformatics/DataHarmonizer/wiki/PHA4GE-SARS-CoV-2-Template	https://github.com/Public-Health-Bioinformatics/DataHarmonizer/releases

1 There are a number of resources that form the PHA4GE SARS-CoV-2 contextual data specification package that are described in the table. The package has been compiled to support user implementation and data sharing, with integration into workflows and new software applications in mind. SOP: standard operating procedure.

## Getting Started—How To Use the Standard

In designing the specification we first considered the goals of data collection and harmonization. Consulted stakeholders believed that the primary priority of standardizing data should be improved support for SARS-CoV-2 genomic surveillance activities and the submission of sequence data and minimal metadata to public repositories. The two most important attributes for tracking transmission from pathogen genomic data are temporal information describing when a sample was collected and spatial information describing where a virus was sampled.

Comparisons of minimal contextual data requirements across different national sequencing efforts, as well as submission requirements for INSDC and GISAID databases, yielded a minimal set of 14 fields that have been annotated as “required” in the specification (colour-coded yellow in the collection template). The required fields, corresponding definitions, and guidance notes are described in Table 3. A number of other fields have been annotated as “strongly recommended” (colour-coded purple in the collection template) for capturing sample collection and processing methods, critical epidemiological information about the host, and acknowledging scientific contributions. Fields colour-coded white are considered optional.

**Table 3:** : Minimal (required) contextual data fields

Field name^1^	Definition	Guidance
specimen collector sample ID	The user-defined name for the sample	Every Sample ID from a single submitter must be unique. It can have any format, but we suggest that you make it concise, unique, and consistent within your laboratory, and as informative as possible
sample collected by	The name of the agency that collected the original sample	The name of the agency should be written out in full (with minor exceptions) and consistent across multiple submissions
sequence submitted by	The name of the agency that generated the sequence	The name of the agency should be written out in full (with minor exceptions) and be consistent across multiple submissions
sample collection date	The date on which the sample was collected	Record the collection date accurately in the template. Required granularity includes year, month, and day. Before sharing these data, ensure that this date is not considered identifiable information. If this date is considered identifiable, it is acceptable to add “jitter” to the collection date by adding or subtracting calendar days. Do not change the collection date in your original records. Alternatively, “received date” may be used as a substitute in the data you share. The date should be provided in ISO 8601 standard format “YYYY-MM-DD”
geo_loc name (country)	Country of origin of the sample	Provide the country name from the pick list in the template
geo_loc name (state/province/region)	State/province/region of origin of the sample	Provide the state/province/region name from the GAZ geography ontology. Search for geography terms at https://www.ebi.ac.uk/ols/ontologies/gaz
Organism	Taxonomic name of the organism	Use “Severe acute respiratory syndrome coronavirus 2”
Isolate	Identifier of the specific isolate	This identifier should be an unique, indexed, alphanumeric ID within your laboratory. If submitted to the INSDC, the “isolate” name is propagated throughout different databases. As such, structure the “isolate” name to be ICTV/INSDC compliant in the following format: “SARS-CoV-2/host/country/sampleID/date”
host (scientific name)	The taxonomic, or scientific name of the host	Common name or scientific name are required if there was a host. Scientific name example: *Homo sapiens*. Select a value from the pick list. If the sample was environmental, put “not applicable.”
host disease	The name of the disease experienced by the host	This field is only required if there was a host. If the host was a human select COVID-19 from the pick list. If the host was asymptomatic, this can be recorded under “host health state details.” “COVID-19” should still be provided if the patient is asymptomatic. If the host is not huma, and the disease state is not known or the host appears healthy, put “not applicable.”
purpose of sequencing	The reason that the sample was sequenced	The reason why a sample was originally collected may differ from the reason why it was selected for sequencing. The reason a sample was sequenced may provide information about potential biases in sequencing strategy. Provide the purpose of sequencing from the pick list in the template. The reason for sample collection should be indicated in the “purpose of sampling” field
sequencing instrument	The model of the sequencing instrument used	Select a sequencing instrument from the pick list provided in the template
consensus sequence software name	The name of software used to generate the consensus sequence	Provide the name of the software used to generate the consensus sequence
consensus sequence software version	The version of the software used to generate the consensus sequence	Provide the version of the software used to generate the consensus sequence

1 Through consultation and consensus, 14 fields were prioritized for SARS-CoV-2 surveillance, which are considered required in the specification. Field names, definitions, and guidance are presented.

Because many contextual data fields are stored in different locations and databases (e.g., LIMS, epidemiology case report forms and databases), a benefit of implementing the PHA4GE collection template is that it enables the capture of these different pieces of information in one place. The collection template also offers pick lists for a variety of fields, e.g., a curated INSDC country list for “geo_loc name (country),” the standardized name of the virus under the “organism” field (i.e., severe acute respiratory coronavirus 2), and a multitude of standardized terms for sample types (anatomical materials and sites, environmental materials and sites, collection devices and methods). The “purpose of sequencing” field provides standardized tags that can be used to highlight sampling strategy criteria (e.g., baseline surveillance [random sampling] or targeted sequencing [non-random sampling]), which are very important for understanding bias when interpreting patterns in sequence data. The pick lists provided are neither exhaustive nor comprehensive but have been curated from current literature representing active sampling and surveillance activities.

If a pick list is missing standardized terms of interest, the reference guide also provides links to different ontology look-up services, enabling users to identify additional standardized terms. The reference guide provides definitions for the fields, additional guidance regarding the structure of the values in the field, and any suggestions for addressing issues pertaining to privacy and identifiability. The curation SOP provides users with step-by-step instructions for populating the template, looking up standardized terms, and how best to structure sample descriptions. The SOP also highlights a number of ethical, practical, and privacy considerations for data sharing.

### Implementation of the PHA4GE specification around the world

The amount of and manner in which the specification is implemented is ultimately at the discretion of the user. To date, versions of the specification are being implemented in the CanCOGeN (Canada) and SPHERES (USA) SARS-CoV-2 sequencing initiatives, the AusTrakka (Australia and New Zealand) data sharing platform [1–3], and by the Global Emerging Pathogens Treatment Consortium (Africa) [63], the African Centre of Excellence for Genomics of Infectious Diseases (ACEGID) in Nigeria [64], the Baobab LIMS [65] at the South African National Bioinformatics Institute (SANBI) [66], and the Latin American Genomics Network [67].

**Figure 2:**
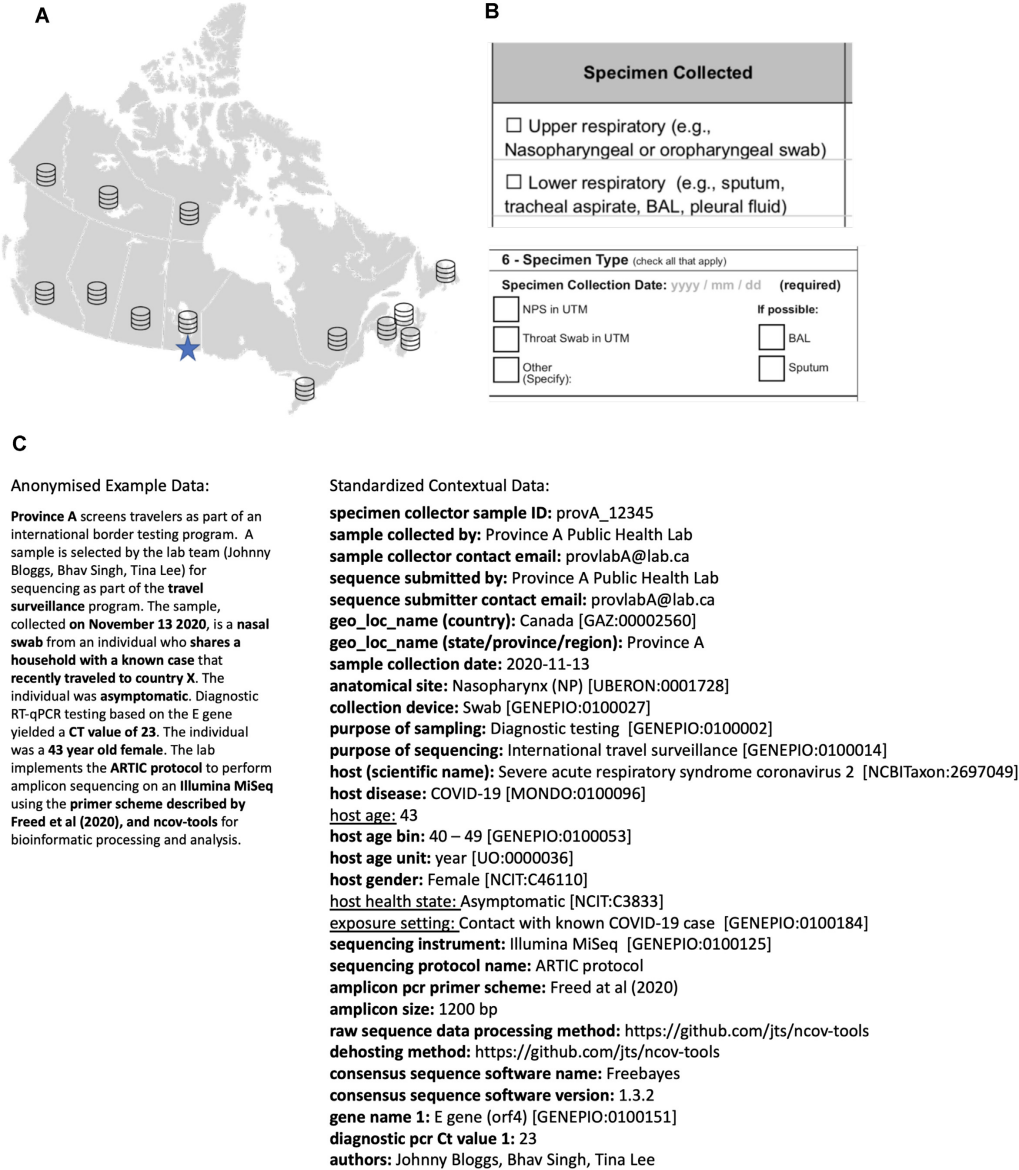
: The PHA4GE specification is being implemented in CanCOGeN to harmonize contextual data across jurisdictions. (A) CanCOGeN is Canada's SARS-CoV-2 national genomic surveillance initiative. Canada has a decentralized health system, with one federal and 13 provincial/territorial public health jurisdictions. Provinces/Territories have authority over how data are collected, stored, and shared. Every Canadian public health jurisdiction uses different collection instruments (e.g., case report forms), different data management systems, and different pipelines and software to perform bioinformatic analyses. Provinces/Territories share sequencing data and accompanying contextual data with the National Microbiology Lab's national SARS-CoV-2 genomics database (starred) according to a version of the PHA4GE specification for national surveillance activities. (B) Excerpts from two different province-specific case collection forms. Sample type information is collected in data collection instruments using different fields, different terms, at different levels of granularity, using abbreviations and formats. BAL: bronchoalveolar lavage; NPS: nasopharyngeal swab; UTM: universal transport medium. (C) An anonymized example of how the standard consistently structures contextual information and how it is being used for data sharing. The contextual data specification provides a wide variety of fields and pick lists of terms. In the example, the full set of standardized information shown would be shared by the province with the national database. Standardized information in boldface would be shared with public repositories; however select data elements (underscored) would be withheld according to jurisdictional data sharing policies. The specification enables users to harmonize and integrate data provenance, sampling strategy criteria, epidemiological information, and methods.

Canada is implementing a version of the PHA4GE specification to harmonize contextual data across all data providers for national SARS-CoV-2 surveillance [5]. Harmonized contextual information is provided by different jurisdictions and stored in the national genomics surveillance database at the Public Health Agency of Canada's National Microbiology Laboratory. A hypothetical worked example is provided to demonstrate how free text information can be structured according to the specification and how subsets of the contextual data can be shared according to jurisdictional policies (Fig. 2).

While the primary use case of the specification is for public health sequencing, the sample collection fields have been developed to enable capture of information for a wide range of sample types, including environmental samples (e.g., swabs of hospital equipment and patient rooms, wastewater samples) and non-human hosts (e.g., wildlife, agricultural animal samples).

## Submitting Data to Public Sequence Repositories

Many existing SARS-CoV-2 sequences have only been deposited in GISAID, with a proportion of submitters also depositing matching raw read data in the INSDC (i.e., NCBI, European Molecular Biology Laboratory–European Bioinformatics Institute [EMBL-EBI], and DNA Data Bank of Japan [DDBJ]). While consensus genomes are widely deposited and used for public surveillance purposes, raw read data are critical for comparing methods and assessing reproducibility, as well as identifying minor variants. Linkage of contextual data to consensus sequences as well as raw data in public repositories is vital.

Within the INSDC, the contextual data are stored as accessioned BioSamples [68] with a consistent set of attribute names and standardized values. BioSamples add value, promote reuse, and enable interoperability of data submitted from laboratories that may only be connected by following the same metadata standard. The INSDC databases have until recently provided a generic pathogen metadata template for the BioSample that is heavily utilized for bacterial genomic surveillance [69]. GISAID uses a different format and data structure for associating metadata primarily for influenza surveillance and now extended to include SARS-CoV-2. The ENA provides a virus metadata checklist (ENA virus pathogen reporting standard checklist) developed as part of the COMPARE project [70], which is very similar to the GISAID submission requirements.

Building on these existing standards, a metadata specification for SARS-CoV-2 genomic surveillance was developed that is broad enough for internal laboratory use while providing mechanisms for mapping/transforming standardized contextual data for public release to INSDC and GISAID. Recently, PHA4GE worked with NCBI to develop a dedicated SARS-CoV-2 BioSample submission package in the NCBI Submission Portal, which incorporates many fields from the PHA4GE standard [71]. The Genomics Standards Consortium will also align its forthcoming “MIxS for SARS-CoV-2” package with this specification. EMBL-EBI will also offer the PHA4GE standard to submitters as one of its validated checklists. Taken together, the PHA4GE specification has already had widespread impact on contextual information data structures around the world.

**Figure 3:**
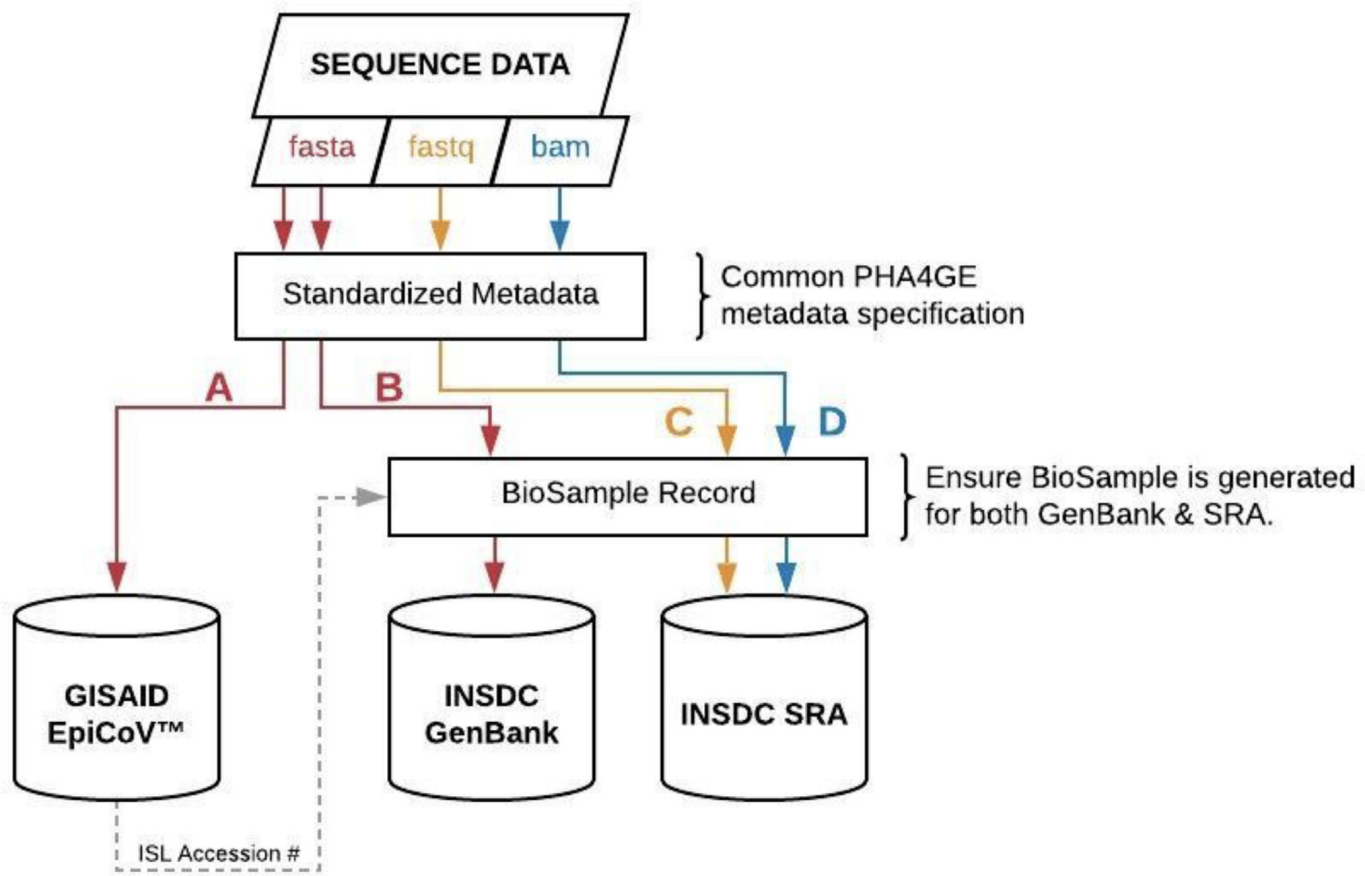
: Overview of how the PHA4GE SARS-CoV-2 contextual data specification can be integrated into public repository submission. The PHA4GE collection template provides a one-stop shop for different data types that are important for global surveillance. The protocols provided as part of the specification package describe how PHA4GE fields can be mapped to different repository submission forms. Consensus sequences (FASTA), accompanied by a subset of PHA4GE fields, can be submitted to the GISAID EpiCoV database (A). Consensus sequences (FASTA) (B) as well as raw/processed data (FASTQ, BAM) (C, D) can be submitted to INSDC databases (e.g., GenBank, SRA) with different subsets of PHA4GE fields as part of a BioSample record. BioSamples are propagated throughout INSDC databases.

The detailed mapping of PHA4GE fields to public repository submission requirements, as well as guidance and advice, are available as supporting documents (see Table 1). We have also provided detailed protocols for data submission to the three participating repositories, GenBank/SRA (NCBI), ENA (EMBL-EBI), and GISAID. An overview of how the PHA4GE specification is integrated into public repository submissions is presented in Fig. 3. PHA4GE recommendations for FAIR SARS-CoV-2 data submissions are as follows:

1. submit raw sequencing data and assembled/consensus genomes to INSDC and GISAID when permitted by jurisdictional data-sharing policies
2. create a BioSample record when submitting to the INSDC using the PHA4GE guidance, populating the mandatory and recommended fields where possible
3. curate public records (sequence data and contextual data), updating them when subsequent information becomes available or retracting if/when records become untrustworthy.

The specification has been used to submit standardized contextual data to different repositories by laboratories and sequencing initiatives globally. A selection of accession numbers for submissions to different repositories is provided in Table 4.

**Table 4:** : A selection of accession numbers of harmonized contextual data records submitted to different public repositories

Data contributor	Repository	Accession No.
African Centre of Excellence for Genomics of Infectious Diseases (Nigeria)	GISAID	EPI_ISL_1 035 827
		EPI_ISL_1 035 826
		EPI_ISL_1 035 825
COVID-19 Genomic Surveillance Regional Network (Latin America)	GISAID	EPI_ISL_2 158 821
		EPI_ISL_2 158 802
		EPI_ISL_2 158 810
COVID-19 Genomic Surveillance Regional Network (Latin America)	EMBL-EBI	SAMEA8968916
Rhode Island Department of Health/Broad Institute (SPHERES)	NCBI	SAMN18306978
Massachusetts General Hospital/Broad Institute (SPHERES)	NCBI	SAMN18309294
Flow Health/Broad Institute (SPHERES)	NCBI	SAMN18308763
New Brunswick Diagnostic Virology Reference Center/Public Health Agency of Canada (CanCOGeN)	NCBI	SAMN16784832
Toronto Invasive Bacterial Diseases Network/McMaster University (CanCOGeN)	NCBI	SAMN17505317
Bat coronavirus phylogeography—Université de La Réunion, UMR Processus Infectieux en Milieu Insulaire Tropical (PIMIT) and Field Museum of Natural History	NCBI	SAMN20400589
		SAMN20400588

## Conclusion

The collective response to the SARS-CoV-2 pandemic has resulted in an unprecedented deployment of genomic surveillance worldwide, bringing together public health agencies, academic research institutions, and industry partners. This unified action provides opportunities to more effectively understand and respond to the pandemic. Yet it also provides an enormous challenge because realizing the full potential of this opportunity will require standardization and harmonization of data collection across these partners. With our SARS-CoV-2 metadata specification we have endeavoured to create a mechanism for promoting consistent, standardized contextual data collection that can be applied broadly. We envision that given the increased uptake, this specification will improve the consistency of collected data, making information reusable by agencies as they continue working towards an increased understanding of SARS-CoV-2 epidemiological and biological characteristics, and harmonizing them such that community-based data-sharing efforts are not excessively burdened. We anticipate that the experience and lessons learned creating the specification package for SARS-CoV-2 will better enable the rapid development and deployment of pathogen-specific standards for public health pathogen genomic surveillance in the future.

## Methods

The PHA4GE SARS-CoV-2 data specification was developed by first comparing existing metadata standards (e.g., MIxS/MIGS, the NIAID/BRC Sample Application Standard) and various sequence repository submission requirements (e.g., GISAID, INSDC), as well as national and international case report forms.

A gap analysis was performed to identify SARS-CoV-2 public health surveillance data elements that were missing in available standards. Fields in existing standards that were deemed to be out of scope were excluded from the specification. Terms for pick lists were sourced from public health documents, the literature, and, when available, various interoperable ontologies (OBO Foundry). The fields and terms from the gap analysis were structured in the collection template (.xlsx). Field definitions, guidance for use, examples, and mappings to various standards were developed as part of the Reference Guides provided in separate tabs in the template workbook. Vocabulary lists were also provided in a separate tab in the template workbook to enable validation and to enable users to add terms to pick lists as needed, according to instructions provided in the curation SOP. The specification was also encoded as a JSON file.

The specification was reviewed by public health, bioinformatics, and data standards experts from different public health agencies, research institutes, and sequencing consortia and adapted according to feedback. Upon request by community members, versioned protocols for public repository submission were created and deposited in protocols.io.

The first version of the specification was made publicly available in August 2020 with a CC-BY 4.0 International attribution license. Iterative improvements were made to a development branch of the specification over the next 10 months as the pandemic evolved, and in response to user feedback and requests. The second major release (2.0) was made publicly available in May 2021. A third major release (3.0) including ontology mappings and the term-level reference guide was made publicly available in December 2021. The PHA4GE template was incorporated into the contextual data harmonization, validation, and transformation tool called The DataHarmonizer through a collaborative effort with the Centre for Infectious Disease Genomics and One Health (Simon Fraser University). Details regarding DataHarmonizer development can be found elsewhere (e.g., [72] and manuscript in preparation (I. Gill et al., in preparation).).

## Availability and Requirements

- Project name: SARS-CoV-2-Contextual-Data-Specification
- Project home page: https://github.com/pha4ge/SARS-CoV-2-Contextual-Data-Specification
- Operating system: Platform independent
- Programming language: Not applicable
- Other requirements: xlsx-compatible spreadsheet software
- License: CC-BY 4.0 International
- RRID:SCR_021378
- biotools:pha4ge_sars-cov-2_contextual_data_specification

## Data Availability

Snapshots of the specification and DataHarmonizer are available in the *GigaScience* GigaDB repository [73].

## Abbreviations

ACEGID: African Center of Excellence for Genomics of Infectious Diseases; CanCOGeN: Canadian COVID Genomics Network; COG-UK: COVID-19 Genomics UK Consortium; COVID-19: coronavirus disease of 2019; EBI: European Bioinformatics Institute; EFO: Experimental Phenotype Ontology; EMBL-EBI: European Molecular Biology Laboratory's European Bioinformatics Institute; ENA: European Nucleotide Archive; FAIR: Findable, Accessible, Interoperable, Reusable; GAZ: Gazetteer Ontology; GenEpiO: Genomic Epidemiology Ontology; GISAID: Global Initiative on Sharing All Influenza Data; HP: Human Phenotype Ontology; INSDC: International Nucleotide Sequence Database Collaboration; INSACOG: Indian SARS-CoV-2 Genomics Consortium; JSON: JavaScript Object Notation; LIMS: Laboratory Information Management System; MIGS: Minimum Information about a Genomic Sequence; MIxS: Minimum Information about any Sequence; MP: Mammalian Phenotype Ontology; NCBI: National Center for Biotechnology Information; NCBITaxon: NCBI Taxonomy Ontology; NCIT: National Cancer Institute Thesaurus; OBI: Ontology for Biological Investigations; OBO Foundry: Open Biological and Biomedical Ontology Foundry; PHA4GE: Public Health Alliance for Genomic Epidemiology; SANBI: South African National Bioinformatics Institute; SARS-CoV-2: severe acute respiratory syndrome coronavirus 2; SOP: standard operating procedure; SPHERES: SARS-CoV-2 Sequencing for Public Health Emergency Response, Epidemiology and Surveillance; SRA: Sequence Read Archive; UBERON: Uber-Anatomy Ontology; UO: Unit Ontology; WHO: World Health Organization.

## Competing Interests

The authors declare that they have no competing interests.

## Funding

The Bill & Melinda Gates Foundation supported the establishment and work of the PHA4GE consortium. A.J.P. and N.F.A. were supported by the Biotechnology and Biological Sciences Research Council (BBSRC), the Quadram Institute Bioscience BBSRC funded Core Capability Grant (project No. BB/CCG1860/1), and the BBSRC Institute Strategic Programme Microbes in the Food Chain BB/R012504/1 and its constituent project BBS/E/F/000PR10352. F.M. was supported by a Donald Hill Family Fellowship in Computer Science. C.I.M. was supported by the Fundação para a Ciência e Tecnologia (grant SFRH/BD/129483/2017). Work by E.J.G., R.C., D.D., and W.W.L.H. was funded by a Genome Canada Bioinformatics and Computational Biology 2017 Grant #286GET and a Genome Canada CanCOGeN grant E09CMA. The work of I.K.M. T.B., and A.J. was supported by the National Center for Biotechnology Information of the National Library of Medicine (NLM), National Institutes of Health.

## Authors’ Contributions

E.J.G.: Conceptualization, Methodology, Investigation, Software, Visualization, Writing—Original Draft Preparation, Validation, Supervision; R.E.T.: Methodology, Investigation, Software, Validation, Writing—Original Draft Preparation; C.I.M.: Methodology, Software, Writing—Review & Editing; A.J.P.: Methodology, Writing—Original Draft Preparation; N.F.A.: Methodology, Software, Validation, Writing—Original Draft Preparation; D.F.: Methodology, Software; F.M.: Writing-Review and Editing, J.C.: Validation, Writing—Review & Editing; D.P.: Validation, Writing—Review & Editing; I.B.O.: Validation, Writing—Review & Editing; D.A.: Software, Validation, Writing—Review & Editing; A.C.: Writing—Review & Editing; A.G.S.: Software, Validation, Writing—Review & Editing; R.C.: Software, Validation; D.D.: Software, Validation; L.S.K.: Validation, Writing—Review & Editing; A.B.: Methodology, Writing—Original Draft Preparation; I.K.M.: Software, Validation, Writing—Review & Editing; T.B.: Software, Validation, Writing—Review & Editing; A.J.: Software, Validation, Writing—Review & Editing; T.R.C.: Validation, Writing—Review & Editing; S.M.N.: Validation, Writing—Review & Editing; A.A.W.: Writing—Review & Editing; P.E.O.: Writing—Review & Editing; G.H.T.: Writing—Review & Editing; S.H.T.: Writing—Review & Editing; A.R.R.: Writing—Review & Editing; B.A.: Writing—Review & Editing; D.M.A.: Writing—Review & Editing; E.H.: Writing—Review & Editing; W.W.L.H.: Writing—Review & Editing; A.T.R.V.: Writing—Review & Editing; D.R.M.: Conceptualization, Methodology, Visualization, Writing—Review & Editing, Funding Acquisition

## Supplementary Material

giac003_GIGA-D-21-00246_Original_Submission

giac003_GIGA-D-21-00246_Revision_1

giac003_Response_to_Reviewer_Comments_Original_Submission

giac003_Reviewer_1_Report_Original_Submission Feng-Biao Guo -- 8/21/2021 Reviewed

giac003_Reviewer_2_Report_Original_Submission Wolfgang Maier -- 9/2/2021 Reviewed

giac003_Reviewer_3_Report_Original_Submission Christopher Hunter, Ph.D. -- 9/9/2021 Reviewed
