# Supplementary material for: Future-proofing and maximizing the utility of metadata: The PHA4GE SARS-CoV-2 contextual data specification package
Source: Gigascience. 2022 Feb 16;11:giac003. doi: 10.1093/gigascience/giac003 (PMC8847733; doi:10.1093/gigascience/giac003)

GigaScience

Future-proofing and maximizing the utility of metadata: The PHA4GE SARS-CoV-2 contextual data specification package

--Manuscript Draft--

**Manuscript Number:**	GIGA-D-21-00246
**Full Title:**	Future-proofing and maximizing the utility of metadata: The PHA4GE SARS-CoV-2 contextual data specification package
**Article Type:**	Technical Note

Funding Information:

Bill and Melinda Gates Foundation	Dr Emma Griffiths
Biotechnology and Biological Sciences Research Council (BB/CCG1860/1)	Dr Andrew J Page
Biotechnology and Biological Sciences Research Council (BB/R012504/1)	Dr Andrew J Page
Biotechnology and Biological Sciences Research Council (BBS/E/F/000PR10352)	Dr Andrew J Page
Donald Hill Family Fellowship	Dr Finlay Maguire
Fundação para a Ciência e a Tecnologia (SFRH/BD/129483/2017)	Ms Catarina Inês Mendes
Genome Canada (286GET)	Dr William WL Hsiao
Genome Canada (E09CMA)	Dr William WL Hsiao
U.S. National Library of Medicine	Dr Ilene Karsch-Mizrachi

Abstract:

The Public Health Alliance for Genomic Epidemiology (PHA4GE) (https://pha4ge.org) is a global coalition that is actively working to establish consensus standards, document and share best practices, improve the availability of critical bioinformatics tools and resources, and advocate for greater openness, interoperability, accessibility and reproducibility in public health microbial bioinformatics. In the face of the current pandemic, PHA4GE has identified a need for a fit-for-purpose, open-source SARS-CoV-2 contextual data standard. As such, we have developed a SARS-CoV-2 contextual data specification package based on harmonisable, publicly available community standards. The specification can be implemented via a collection template, as well as an array of protocols and tools to support both the harmonisation and submission of sequence data and contextual information to public biorepositories. Well-structured, rich contextual data adds value, promotes reuse, and enables aggregation and integration of disparate data sets. Adoption of the proposed standard and practices will better enable interoperability between datasets and systems, improve the consistency and utility of generated data, and ultimately facilitate novel insights and discoveries in SARS-CoV-2 and COVID-19. The package is now supported by the National Center for Biotechnology (NCBI)'s BioSample database.

**Corresponding Author:**	Emma Griffiths<br>Simon Fraser University<br>Vancouver, British Columbia CANADA
**Corresponding Author Secondary Information:**	
**Corresponding Author's Institution:**	Simon Fraser University
**Corresponding Author's Secondary Institution:**	
**First Author:**	Emma Griffiths
**First Author Secondary Information:**	

Powered by Editorial Manager® and ProduXion Manager® from Aries Systems Corporation

**Order of Authors:**	Emma Griffiths
	Ruth E Timme
	Catarina Inês Mendes
	Andrew J Page
	Nabil-Fareed Alikhan
	Dan Fornika
	Finlay Maguire
	Josefina Campos
	Daniel Park
	Idowu B Olawoye
	Paul E Oluniyi
	Dominique Anderson
	Alan Christoffels
	Anders Gonçalves da Silva
	Rhiannon Cameron
	Damion Dooley
	Lee S Katz
	Allison Black
	Ilene Karsch-Mizrachi
	Tanya Barrett
	Anjanette Johnston
	Thomas R Connor
	Samuel M Nicholls
	Adam A Witney
	Gregory H Tyson
	Simon H Tausch
	Amogelang R Raphenya
	Brian Alcock
	David M Aanensen
	Emma Hodcroft
	William WL Hsiao
	Ana Tereza R Vasconcelos
	Duncan R MacCannell
**Order of Authors Secondary Information:**	
**Additional Information:**	
**Question**	**Response**
Are you submitting this manuscript to a special series or article collection?	No
**Experimental design and statistics**	Yes

Powered by Editorial Manager® and ProduXion Manager® from Aries Systems Corporation

Full details of the experimental design and statistical methods used should be given in the Methods section, as detailed in our Minimum Standards Reporting Checklist. Information essential to interpreting the data presented should be made available in the figure legends.<br><br>Have you included all the information requested in your manuscript?	
**Resources**<br><br>A description of all resources used, including antibodies, cell lines, animals and software tools, with enough information to allow them to be uniquely identified, should be included in the Methods section. Authors are strongly encouraged to cite Research Resource Identifiers (RRIDs) for antibodies, model organisms and tools, where possible.<br><br>Have you included the information requested as detailed in our Minimum Standards Reporting Checklist?	Yes
**Availability of data and materials**<br><br>All datasets and code on which the conclusions of the paper rely must be either included in your submission or deposited in publicly available repositories (where available and ethically appropriate), referencing such data using a unique identifier in the references and in the "Availability of Data and Materials" section of your manuscript.<br><br>Have you have met the above requirement as detailed in our Minimum Standards Reporting Checklist?	Yes

Powered by Editorial Manager® and ProduXion Manager® from Aries Systems Corporation

Manuscript

Click here to access/download;Manuscript;PHA4GE SC2 Manuscript_Gigascience_2021.docx

Future-proofing and maximizing the utility of metadata: The PHA4GE SARS-CoV-2 contextual data specification package

Authors

Emma J Griffiths [1*], Ruth E Timme [2], Catarina Inês Mendes [3], Andrew J Page [4], Nabil-Fareed Alikhan [4], Dan Fornika [5], Finlay Maguire [6], Josefina Campos [7], Daniel Park [8], Idowu B Olawoye [9, 10], Paul E Oluniyi [9, 10], Dominique Anderson [11], Alan Christoffels [11], Anders Gonçalves da Silva [12], Rhiannon Cameron [1], Damion Dooley [1], Lee S Katz [13], Allison Black [14], Ilene Karsch-Mizrachi [15], Tanya Barrett [15], Anjanette Johnston [15], Thomas R Connor [16, 17], Samuel M Nicholls [18], Adam A Witney [19], , Gregory H Tyson [20], Simon H Tausch [21], Amogelang R Raphenya [22], Brian Alcock [22], David M Aanensen [23, 24], Emma Hodcroft [25], William WL Hsiao [1, 5, 26], Ana Tereza R Vasconcelos [27], and Duncan R MacCannell [28], on behalf of the Public Health Alliance for Genomic Epidemiology (PHA4GE) consortium.

Affiliations

1 Faculty of Health Sciences, Simon Fraser University, Burnaby, British Columbia, Canada.
2 Center for Food Safety and Applied Nutrition, U.S. Food and Drug Administration, College Park, MD, USA.
3 Instituto de Microbiologia, Instituto de Medicina Molecular, Faculdade de Medicina, Universidade de Lisboa, Lisboa, Portugal.
4 Quadram Institute Bioscience, Norwich, Norfolk, UK.
5 BC Centre for Disease Control Public Health Laboratory, Vancouver, Canada.
6 Faculty of Computer Science, Dalhousie University, Halifax, Canada.
7 INEI-ANLIS "Dr Carlos G. Malbrán", Buenos Aires, Argentina.
8 The Broad Institute of MIT and Harvard, Cambridge, MA, USA.
9 African Center of Excellence for Genomics of Infectious Diseases (ACEGID), Redeemer's University, Ede, Osun State, Nigeria.
10 Department of Biological Sciences, College of Natural Sciences, Redeemer's University, Ede, Osun State, Nigeria
11 South African Medical Research Council Bioinformatics Unit, South African National Bioinformatics Institute, University of the Western Cape, Bellville, South Africa.
12 Microbiological Diagnostic Unit Public Health Laboratory, The Peter Doherty Institute for Infection and Immunity, The University of Melbourne, Melbourne, Victoria, Australia
13 Center for Food Safety, University of Georgia, Georgia, USA
14 Department of Epidemiology, University of Washington, Washington, USA.
15 National Center for Biotechnology Information, National Library of Medicine, National Institutes of Health, Bethesda, MD, USA
16 Organisms and Environment Division, School of Biosciences, Cardiff University, Cardiff, Wales, UK.

17 Public Health Wales, University Hospital of Wales, Cardiff, UK
18 University of Birmingham, Birmingham, UK
19 Institute for Infection and Immunity, St George's, University of London, London, UK
20 Center for Veterinary Medicine, U.S. Food and Drug Administration, Laurel, Maryland, USA
21 Department of Biological Safety, German Federal Institute for Risk Assessment, Berlin, Germany
22 Department of Biochemistry and Biomedical Sciences and the Michael G. DeGroote Institute for Infectious Disease Research, McMaster University, Hamilton, Ontario, Canada
23 Centre for Genomic Pathogen Surveillance, Wellcome Genome Campus, Cambridge, UK
24 The Big Data Institute, Li Ka Shing Centre for Health Information and Discovery, Nuffield Department of Medicine, University of Oxford, Oxford, UK.
25 Biozentrum, University of Basel, Basel, Switzerland & Swiss Institute of Bioinformatics, Lausanne, Switzerland
26 Department of Pathology and Laboratory Medicine, University of British Columbia, Vancouver, Canada
27 Bioinformatics Laboratory National Laboratory of Scientific Computation LNCC/MCTI, Rio de Janeiro, Brazil
28 National Center for Emerging and Zoonotic Infectious Diseases, Centers for Disease Control and Prevention, Georgia, USA

*Corresponding author: emma_griffiths@sfu.ca

Author Email Addresses

Emma J Griffiths: ega12@sfu.ca
Ruth E Timme: ruth.timme@fda.hhs.gov
Catarina Inês Mendes: cimendes@medicina.ulisboa.pt
Andrew J Page: Andrew.Page@quadram.ac.uk
Nabil-Fareed Alikhan: nabil-fareed.alikhan@quadram.ac.uk
Dan Fornika: dan.fornika@bccdc.ca
Finlay Maguire: finlaymaguire@gmail.com
Josefina Campos: jocampos05@gmail.com
Daniel Park: dpark@broadinstitute.org
Idowu B Olawoye: olawoyei0303@run.edu.ng
Paul E Oluniyi: oluniyip@run.edu.ng
Dominique Anderson: dominique@sanbi.ac.za
Alan Christoffels: alan@sanbi.ac.za
Anders Gonçalves da Silva: anders.goncalves@unimelb.edu.au
Rhiannon Cameron: cmrn.rhi@gmail.com
Damion Dooley: damion_dooley@sfu.ca
Lee S Katz: gzu2@cdc.gov
Allison Black: black.alli@gmail.com
Ilene Karsch-Mizrachi: mizrachi@ncbi.nlm.nih.gov
Tanya Barrett: barrett@ncbi.nlm.nih.gov
Anjanette Johnston: johnston@ncbi.nlm.nih.gov
Thomas R Connor: connortr@cardiff.ac.uk
Samuel M Nicholls: sam@samnicholls.net

Adam A Witney: awitney@sgul.ac.uk
Gregory H Tyson: Gregory.Tyson@fda.hhs.gov
Simon H Tausch: simon.tausch@bfr.bund.de
Amogelang R Raphenya: raphenar@mcmaster.ca
Brian Alcock: brian.alcock@gmail.com
David M Aanensen: david.aanensen@bdi.ox.ac.uk
Emma Hodcroft: emmahodcroft@gmail.com
William WL Hsiao: wwhsiao@sfu.ca
Ana Tereza R Vasconcelos: atrv@lncc.br
Duncan R MacCannell: fms2@cdc.gov

Abstract

The Public Health Alliance for Genomic Epidemiology (PHA4GE) (https://pha4ge.org) is a global coalition that is actively working to establish consensus standards, document and share best practices, improve the availability of critical bioinformatics tools and resources, and advocate for greater openness, interoperability, accessibility and reproducibility in public health microbial bioinformatics. In the face of the current pandemic, PHA4GE has identified a need for a fit-for-purpose, open-source SARS-CoV-2 contextual data standard. As such, we have developed a SARS-CoV-2 contextual data specification package based on harmonisable, publicly available community standards. The specification can be implemented via a collection template, as well as an array of protocols and tools to support both the harmonisation and submission of sequence data and contextual information to public biorepositories. Well-structured, rich contextual data adds value, promotes reuse, and enables aggregation and integration of disparate data sets. Adoption of the proposed standard and practices will better enable interoperability between datasets and systems, improve the consistency and utility of generated data, and ultimately facilitate novel insights and discoveries in SARS-CoV-2 and COVID-19. The package is now supported by the National Center for Biotechnology (NCBI)'s BioSample database.

Keywords: genomics, metadata, SARS-CoV-2, bioinformatics, data standards

Findings

The importance of contextual data for interpreting SARS-CoV-2 sequences

First identified in late 2019 in Wuhan, China, the SARS-CoV-2 virus has now spread to virtually every country and territory in the world, resulting in millions of

confirmed cases, and deaths, globally. [1], [2]. Understanding, monitoring and preventing transmission, as well as the development of vaccines and effective therapeutic options, have been primary goals of the public health response to SARS-CoV-2.

Tracking the spread and evolution of the virus at global, national and local scales has been aided by the analysis of viral genome sequence data alongside SARS-CoV-2 epidemiology. Large scale sequencing efforts are often formalised as consortia across the world, including the COG-UK in the UK [3], SPHERES in the USA [4], CanCOGeN in Canada [5], Latin American Genomics SARS-CoV-2 Network [6], [7], 2019nCoVR in China [8], the South Africa NGS Genomic Surveillance Network [9], AusTrakka in Australia and New Zealand [10], and INSACOG in India [11]. In addition to these initiatives, many agencies, universities and hospital laboratories around the world are also sequencing and sharing sequence data at an unprecedented pace. Deposition of these sequences into public repositories such as the Global Initiative on Sharing All Influenza Data (GISAID) and the International Nucleotide Sequence Database Collaboration (INSDC) has enabled rapid global sharing of data [12], [13]. At the time of writing, 174 countries had undertaken open sequencing initiatives (GISAID accessed 2021-06-23) depositing 2,057,675 sequences which are being reused and analysed on a massive scale. The open data sharing paradigm has had tremendous success in the genomic epidemiology of foodborne pathogens [14], [15], and has the potential to reveal a deeper understanding of SARS-CoV-2 origin, pathogenicity, and basic biology when submissions from environmental samples and wild hosts are included alongside human clinical samples [16].

SARS-CoV-2 sequencing, analysis, and open sharing have played a crucial role in a number of developments during the pandemic, such as dispelling misinformation about the origins of the virus [17], the identification and surveillance of variants of concern [18], [19], the improvement of diagnostic performance and rapid testing [20]–[22], and the development of vaccines which are currently being distributed in the largest global vaccination program the world has ever seen [23]. Viral genomic sequences are also being used to understand transmission and reinfection events [24] as well to monitor the prevalence and diversity of lineages during different exposure events and in different settings e.g. animal reservoirs [25], long-term care facilities [26]–[28], healthcare and other work sites [29]–[33], conferences and other public gatherings [34], as well before and after public health responses (e.g. border controls and travel restrictions, lockdowns and quarantines, vaccination, etc.), through successive waves of infections [35]–[46]. However, it is critical to note that public health sequence data is of limited value without accompanying contextual metadata.

Contextual data consists of sample metadata (e.g., collection date, sample type, geographical location of sample collection), as well as laboratory (e.g., date and location testing, cycle threshold (CT) values), clinical outcomes (e.g., hospitalization, death, recovery), epidemiological (e.g., age, gender, exposures, vaccination status) and methods (e.g., sampling, sequencing, bioinformatics) that enable the interpretation sequence data (e.g., previous examples). High-quality contextual data is also crucial for quality control. For example, detecting systematic batch effect errors related to certain sequencing centres and methods can help evaluate which variants represent real, circulating viruses, as opposed to artifacts of sample handling or sequencing which may arise due to different aspects experimental design, laboratory procedures, bioinformatics processing, and applied quality control thresholds [47]–[49].

Good data stewardship practices are not only critical for auditability and reproducibility, but for posterity - documenting critical information about samples, methods, risk factors and outcomes etc., can help future-proof information used to build a roadmap for dealing with future public health crises. Contextual data, however, is often collected on a project-specific basis according to local needs and reporting requirements which results in the collection of different data types at different levels of granularity, with different meanings and implicit bias of variables and attributes. Furthermore, the information is often collected as free text, or if structured, according to organization or initiative-specific data dictionaries, using different fields, terms, formats, abbreviations, and jargon.

The variability in the way information is encoded in private databases tends to propagate to public repositories, which makes the information more difficult to interpret and to use. There are different existing standards that can be used to structure contextual data, like minimum information checklists (MIxS [50], MIGS [51], the NIAID/BRC Project and Sample Application Standard [52]) and various interoperable ontologies (OBO Foundry [53]), which make information easier to aggregate and reuse for different types of analyses. However, these attribute packages and metadata standards developed by different organizations are usually scoped to cover as many use cases and pathogens as possible, and as such, can include fields of information not applicable to SARS-CoV-2, or that may be subject to privacy concerns, or exclude fields commonly used in public health surveillance and investigations. As different types of contextual data are subject to different ethical, practical and privacy concerns, not all components of existing standards are immediately or widely collectable and shareable. As a result, the range of generic metadata standards being applied to SARS-CoV-2 data presents challenges for data harmonization [54] and analysis critical for fighting the disease and ending the pandemic.

In light of these challenges, PHA4GE has identified a need for a fit-for-purpose, open-source SARS-CoV-2 contextual data specification which can be used to consistently structure information as part of good data management practices and for data sharing with trusted partners and/or public repositories. The specification was developed by consensus among domain experts, and incorporates existing community standards with an emphasis on SARS-CoV-2 public health needs and ensuring privacy while maximizing information content and interoperability across datasets and databases to better enable analyses to fight COVID-19. The specification package also contains a number of accompanying materials such as standard operating procedures, tools, a reference guide, and repository submission protocols (protocols.io) to help put the standard into practice.

SARS-CoV-2 Contextual Data Specification: The Framework

The purpose of the PHA4GE SARS-CoV-2 specification is to provide a mechanism for consistent structure, collection and formatting of fields and values containing SARS-CoV-2 contextual data. We emphasize that the purpose of this specification is not to force data sharing, but rather to provide a framework to structure data consistently across disparate laboratory and epidemiological databases so that they can be harmonized for different uses (Figure 1). Data sharing is just one use case and can involve sharing between divisions within a single agency, sharing between partners based on memorandums of understanding, or submission to public repositories.

The PHA4GE SARS-CoV-2 contextual data specification was created through broad consultation with representatives from public health laboratories, research institutes and universities in 11 countries (Argentina, Australia, Brazil, Canada, Germany, Nigeria, Portugal, South Africa, Switzerland, the United Kingdom, the United States of America) who are involved with the SARS-CoV-2 genome sequencing and analysis efforts at various scales. Based on this consultation and consensus, the specification contains different fields covering a wide array of data types described in Box 1 (Figure 1). The specification attempts to harmonize different data standards (INSDC, GISAID, MIxS, MIGS, Sample Application Standard) by reusing fields or mapping to fields, as much as possible. As PHA4GE embraces FAIR data stewardship principles (Findability, Accessibility, Interoperability and Reuse of digital assets), we strived to implement FAIR principles in the design and implementation of the specification for data management and data sharing. At their core, these principles emphasize machine-actionability and consistency of data, and are critical for dealing with the volume and complexity of genomic sequence and contextual data. Principles of FAIR data stewardship that have been implemented include improving machine-actionability of data by using a formal, accessible, shared, and broadly applicable

language for knowledge representation, reusing existing standards and ontology-based vocabulary to increase interoperability, providing a data usage license, capturing data provenance, and making all resources open, free and widely accessible.

The versioned specification is available as a contextual data collection template (.xlsx) and in machine-amenable JSON format from GitHub (v 2.0.0 - https://github.com/pha4ge/SARS-CoV-2-Contextual-Data-Specification) [55]. The collection template also offers standardized terms for a number of fields in the form of pick lists. The fields are colour-coded to indicate required (yellow), strongly recommended (purple) or optional status (white). Fields useful for surveillance were prioritized as required. Formats for data elements like dates are also prescribed according to international standards (e.g., dates should be formatted according to ISO8601).

The template is also supported by several materials such as a Reference Guide (available as a tab in the collection template Excel workbook), which provides definitions and field-level guidance and examples of usage [55]. The Reference Guide also provides mapping of PHA4GE fields to existing contextual data standards, highlighting public health and SARS-CoV-2-specific fields that were missing as well as fields in those other standards that were considered out of scope.

The Open Biological and Biomedical Ontology (OBO) Foundry is a community of researchers that use a prescribed set of principles and practices to develop a wide range of interoperable ontologies focused on the life sciences [56]. Fields and terms in the specification are currently being mapped to existing OBO Foundry ontology terms, and ontology terms and axioms for vocabulary that currently do not exist are being developed and will be made available in different application and domain-specific ontologies within The Foundry (i.e., GenEpiO, OBI, GAZ, UO, HP, MP, EFO, UBERON, NCIT, NCBITaxon, etc.). We anticipate that these freely available, open-source resources will be of use to the COVID-19 research community (manuscript in preparation).

Protocols have also been created and are openly available on protocols.io [57], including a curation Standard Operating Procedure (SOP) containing instructions for using the collection template as well as guidance for a number of privacy and practical concerns. A series of versioned SARS-CoV-2 sequence and contextual data submission protocols and accompanying instructional videos for how to prepare submissions and navigate through the various submission portals for GISAID, NCBI and EMBL-EBI are also provided.

A mapping file indicating which PHA4GE fields correspond to which fields within the different repository submission forms has also been provided to facilitate data transformations for submissions. Such transformations can be automated using a contextual data harmonization application called the DataHarmonizer [58]. PHA4GE has worked with the developers of the DataHarmonizer to offer the fields and terms of the PHA4GE standard as a template in the tool. Users can standardize and validate entered data and export it as GISAID and NCBI-ready submission forms (BioSample, SRA, GenBank and GenBank source modifier forms). A table outlining the different specification package materials can be found in Table 1.

Table 1: Resources that form the PHA4GE SARS-CoV-2 contextual data specification package [55]

**Resource[1]**	**Description**	**Link**
Collection template and controlled vocabulary pick lists	Spreadsheet-based collection form containing different fields (identifiers and accessions, sample collection and processing, host information, host exposure, vaccination and reinfection information, lineage and variant information, sequencing, bioinformatics and QC metrics, diagnostic testing information, author acknowledgements). Fields are colour-coded to indicate required, recommended or optional status. Many fields offer pick lists of controlled vocabulary. Vocabulary lists are also available in a separate tab.	https://github.com/pha4ge/SARS-CoV-2-Contextual-Data-Specification/raw/master/PHA4GE%20SARS-CoV-2%20Contextual%20Data%20Template.xlsx
Reference guide	Field definitions, guidance, and examples are provided as a separate tab in the collection template .xlsx file.	https://github.com/pha4ge/SARS-CoV-2-Contextual-Data-Specification/raw/master/PHA4GE%20SARS-CoV-2%20Contextual%20Data%20Template.xlsx
Curation protocol on protocols.io	Step-by-step instructions for using the collection template are provided in a standard operating procedure (SOP). Ethical, practical, and privacy considerations are also discussed. Examples and instructions for structuring sample descriptions as well as sourcing additional standardized terms (outside those provided in pick lists) are also discussed.	https://www.protocols.io/view/pha4ge-contextual-metadata-sop-btpznmp6
Mapping file of PHA4GE fields to metadata standards	PHA4GE fields are mapped to existing metadata standards such as the Sample Application Standard, MIxS 5.0, and the MIGS Virus Host-associated attribute package. Mappings are	https://github.com/pha4ge/SARS-CoV-2-Contextual-Data-Specification/raw/master/PH

	available in the Reference guide tab. Mappings highlight which fields of these standards are considered useful for SARS-CoV-2 public health surveillance and investigations, and which fields are considered out of scope.	A4GE%20SARS-CoV-2%20Contextual%20Data%20Template.xlsx
Mapping file of PHA4GE fields to EMBL-EBI, NCBI and GISAID submission requirements	Many PHA4GE fields have been sourced from public repository submission requirements. The different repositories have different requirements and field names. Repository submission fields have been mapped to PHA4GE fields to demonstrate equivalencies and divergences.	https://github.com/pha4ge/SARS-CoV-2-Contextual-Data-Specification/raw/master/PHA4GE%20to%20Sequence%20Repository%20Field%20Mappings.xlsx
Data submission protocol (NCBI) on protocols.io	The SARS-CoV-2 submission protocol for NCBI provides step-by-step instructions and recommendations aimed at improving interoperability and consistency of submitted data.	https://www.protocols.io/view/sars-cov-2-ncbi-submission-workflow-guidance-for-s-bsypnfvn
Data submission protocol (EMBL-EBI) on protocols.io	The SARS-CoV-2 submission protocol for ENA provides step-by-step instructions and recommendations aimed at improving interoperability and consistency of submitted data.	https://www.protocols.io/view/sars-cov-2-ena-submission-workflow-guidance-for-st-buqnnvve
Data submission protocol (GISAID) on protocols.io	The SARS-CoV-2 submission protocol for GISAID provides step-by-step instructions and recommendations aimed at improving interoperability and consistency of submitted data.	https://www.protocols.io/view/sars-cov2-gisaid-submission-protocol-bumknu4w
JSON structure of PHA4GE specification	A JSON structure of the PHA4GE specification has been provided for easier integration into software applications.	https://raw.githubusercontent.com/pha4ge/SARS-CoV-2-Contextual-Data-Specification/master/PHA4GE_SARS-CoV-2_Contextual_Data_Schema.json
PHA4GE template in the DataHarmonizer	Javascript application enabling standardized data entry, validation and export of contextual data as submission-ready forms for GISAID and NCBI. The SOP for using the software can be found at https://github.com/Public-Health-Bioinformatics/DataHarmonizer/wiki/PHA4GE-SARS-CoV-2-Template	https://github.com/Public-Health-Bioinformatics/DataHarmonizer/releases

[1]There are a number of resources that form the PHA4GE SARS-CoV-2 contextual data specification package which are described in the table. The package has been compiled to support user implementation and data sharing, with integration into workflows and new software applications in mind.

Getting Started - How To Use The Standard

In designing the specification we first considered the goals of data collection and harmonization. Consulted stakeholders felt that the primary priority of standardizing data should be improved support for SARS-CoV-2 genomic surveillance activities and the submission of sequence data and minimal metadata to public repositories. The two most important attributes for tracking transmission from pathogen genomic data are temporal information describing when a sample was collected and spatial information describing where a virus was sampled.

Comparisons of minimal contextual data requirements across different national sequencing efforts, as well as submission requirements for INSDC and GISAID databases, yielded a minimal set of 15 fields which have been annotated as “required” in the specification (colour-coded yellow in the collection template, see Table 1). The fields, corresponding definitions, and guidance notes are described in Table 2. A number of other fields have been annotated as “strongly recommended” (colour-coded purple in the collection template) for capturing sample collection and processing methods, critical epidemiological information about the host, and acknowledging scientific contributions. Fields colour-coded white are considered optional.

Table 2: Minimal (required) contextual data fields

**Field Name**[1]	**Definition**	**Guidance**
specimen collector sample ID	The user-defined name for the sample.	Every Sample ID from a single submitter must be unique. It can have any format, but we suggest that you make it concise, unique and consistent within your lab, and as informative as possible.
sample collected by	The name of the agency that collected the original sample.	The name of the agency should be written out in full, (with minor exceptions) and consistent across multiple submissions.
sequence submitted by	The name of the agency that generated the sequence.	The name of the agency should be written out in full, (with minor exceptions) and be consistent across multiple submissions.
sample collection date	The date on which the sample was collected.	Record the collection date accurately in the template. Required granularity includes year, month and day. Before sharing this data, ensure this date is not considered identifiable information. If this date is considered identifiable, it is acceptable to add "jitter" to the collection date by adding or subtracting calendar days. Do not change the collection date in your original records. Alternatively,” received date” may be used as a substitute in the data you

		share. The date should be provided in ISO 8601 standard format "YYYY-MM-DD".
geo_loc name (country)	Country of origin of the sample.	Provide the country name from the pick list in the template.
geo_loc name (state/province/region)	State/province/region of origin of the sample.	Provide the state/province/region name from the GAZ geography ontology. Search for geography terms here: https://www.ebi.ac.uk/ols/ontologies/gaz
organism	Taxonomic name of the organism.	Use “Severe acute respiratory syndrome coronavirus 2”
isolate	Identifier of the specific isolate.	This identifier should be an unique, indexed, alpha-numeric ID within your laboratory. If submitted to the INSDC, the "isolate" name is propagated throughout different databases. As such, structure the "isolate" name to be ICTV/INSDC compliant in the following format: "SARS-CoV-2/host/country/sampleID/date"
host (scientific name)	The taxonomic, or scientific name of the host.	Common name or scientific name are required if there was a host. Scientific name examples e.g., Homo sapiens. Select a value from the pick list. If the sample was environmental, put "not applicable".
host disease	The name of the disease experienced by the host.	This field is only required if there was a host. If the host was a human select COVID-19 from the pick list. If the host was asymptomatic, this can be recorded under “host health state details”. "COVID-19" should still be provided if the patient is asymptomatic. If the host is not human, and the disease state is not known or the host appears healthy, put “not applicable”.
purpose of sequencing	The reason that the sample was sequenced.	The reason why a sample was originally collected may differ from the reason why it was selected for sequencing. The reason a sample was sequenced may provide information about potential biases in sequencing strategy. Provide the purpose of sequencing from the picklist in the template. The reason for sample collection should be indicated in the "purpose of sampling" field.
purpose of sequencing details	The description of why the sample was sequenced providing specific details.	Provide an expanded description of why the sample was sequenced using free text. The description may include the importance of the sequences for a

		particular public health investigation/surveillance activity/research question. Suggested standardized descriptions include: Screened for S gene target failure (S dropout), Screened for mink variants, Screened for B.1.1.7 variant, Screened for B.1.135 variant, Screened for P.1 variant, Screened due to travel history, Screened due to close contact with infected individual, Assessing public health control measures, Determining early introductions and spread, Investigating airline-related exposures, Investigating temporary foreign worker, Investigating remote regions, Investigating health care workers, Investigating schools/universities, Investigating reinfection.
sequencing instrument	The model of the sequencing instrument used.	Select a sequencing instrument from the picklist provided in the template.
consensus sequence software name	The name of software used to generate the consensus sequence.	Provide the name of the software used to generate the consensus sequence.
consensus sequence software version	The version of the software used to generate the consensus sequence.	Provide the version of the software used to generate the consensus sequence.

[1] Through consultation and consensus, fifteen fields were prioritized for SARS-CoV-2 surveillance, which are considered required in the specification. Field names, definitions, and guidance are presented.

As many contextual data fields are stored in different locations and databases (e.g., LIMS, epidemiology case report forms and databases), a benefit of implementing the PHA4GE collection template is that it enables the capture of these different pieces of information in one place. The collection template also offers picklists for a variety of fields e.g., a curated INSDC country list for “geo_loc name (country)”, the standardised name of the virus under the “organism” field (i.e., Severe acute respiratory coronavirus 2), and a multitude of standardised terms for sample types (anatomical materials and sites, environmental materials and sites, collection devices and methods). The “purpose of sequencing” field provides standardized tags which can be used to highlight sampling strategy criteria (e.g., baseline surveillance (random sampling), targeted sequencing (non-random sampling), which are very important for understanding bias when interpreting patterns in sequence data. The picklists provided are neither exhaustive, nor comprehensive, but have been curated from current literature representing active sampling and surveillance activities.

If a pick list is missing standardised terms of interest, the reference guide also provides links to different ontology look-up services enabling users to identify additional standardized terms. The reference guide provides definitions for the fields, additional guidance regarding the structure of the values in the field, and any suggestions for addressing issues pertaining to privacy and identifiability. The curation SOP provides users with step-by-step instructions for populating the template, looking up standardized terms, and how best to structure sample descriptions. The SOP also highlights a number of ethical, practical, and privacy considerations for data sharing.

Implementation of the PHA4GE specification around the world

The amount of, and manner in which the specification is implemented is ultimately at the discretion of the user. To date, versions of the specification are being implemented in the CanCOGeN (Canada) and SPHERES (USA) SARS-CoV-2 sequencing initiatives, the AusTrakka (Australia and New Zealand) data sharing platform [10], by the Global Emerging Pathogens Treatment Consortium (Africa)[59], the African Centre of Excellence for Genomics of Infectious Diseases (ACEGID) in Nigeria [60], the Baobab LIMS [61] at the South African National Bioinformatics Institute (SANBI) [62], and the Latin American Genomics Network [63].

Canada is implementing a version of the PHA4GE specification to harmonise contextual data across all data providers for national SARS-CoV-2 surveillance [5]. Harmonised contextual information is provided by different jurisdictions, and stored in the national genomics surveillance database at the Public Health Agency of Canada’s National Microbiology Laboratory. A worked example is provided to demonstrate how free text information can be structured according to the specification, and how subsets of the contextual data can be shared according to jurisdictional policies (Figure 2).

While the primary use case of the specification is for clinical sequencing, the sample collection fields have been developed to enable capture of information for a wide range of sample types, including environmental samples (e.g., swabs of hospital equipment and patient rooms, wastewater samples) and non-human hosts (e.g., wildlife, agricultural animal samples).

Submitting Data to Public Sequence Repositories

Most existing SARS-CoV-2 sequences have only been deposited in GISAID, with a proportion of submitters also depositing matching raw read data in the INSDC (i.e., National Center for Biotechnology Information (NCBI), European Molecular Biology Laboratory-European Bioinformatics Institute (EMBL-EBI) and DNA Data Bank of Japan (DDBJ)). While consensus genomes are widely deposited and used for public surveillance purposes, raw read data is critical for comparing methods, assessing

reproducibility, as well as identifying minor variants. Linkage of contextual data to consensus sequences as well as raw data in public repositories is vital.

Within the INSDC, the contextual data is stored as accessioned BioSamples [64] with a consistent set of attribute names and standardized values. BioSamples add value, promote reuse, and enable interoperability of data submitted from laboratories that may only be connected by following the same metadata standard. The INSDC databases have until recently provided a generic pathogen metadata template for the BioSample that is heavily utilized for bacterial genomic surveillance [65]. GISAID uses a different format and data structure for associating metadata primarily for influenza surveillance and now extended to include SARS-CoV-2. The ENA provides a virus metadata checklist (ENA virus pathogen reporting standard checklist) developed as part of the COMPARE project [66], which is very similar to the GISAID submission requirements.

Building off of these existing standards, a metadata specification for SARS-CoV-2 genomic surveillance was developed that is broad enough for internal laboratory use while providing mechanisms for mapping/transforming standardized contextual data for public release to INSDC and GISAID. Recently, PHA4GE worked with NCBI to develop a dedicated SARS-CoV-2 BioSample submission package in the NCBI Submission Portal, which incorporates many fields from the PHA4GE standard [67]. The Genomics Standards Consortium will also align its forthcoming “MIxS for SARS-CoV-2” package with this specification. EMBL-EBI will also offer the PHA4GE standard to submitters as one of its validated checklists. Taken together, the PHA4GE specification has already had widespread impact on contextual information data structures around the world.

The detailed mapping of PHA4GE fields to public repository submission requirements, as well as guidance and advice, are available as supporting documents (see Table 1). We have also provided detailed protocols for data submission to the three participating repositories, GenBank/SRA (NCBI), ENA (EMBL-EBI), and GISAID. An overview of how the PHA4GE specification is integrated into public repository submissions is presented in Figure 3. PHA4GE recommendations for FAIR SARS-CoV-2 data submissions are as follows:

1. submit raw sequencing data and assembled/consensus genomes to INSDC and GISAID when permitted by jurisdictional data sharing policies
2. create a BioSample record when submitting to the INSDC using the PHA4GE guidance, populating the mandatory and recommended fields where possible
3. curate public records (sequence data and contextual data), updating them when subsequent information becomes available or retracting if/when records become untrustworthy.

The specification has been used to submit standardized contextual data to different repositories by labs and sequencing initiatives globally. A selection of accession numbers for submissions to different repositories is provided below (Table 3).

Table 3: A selection of accession numbers of harmonized contextual data records submitted to different public repositories

Data Contributor	Repository Name	Accession Number
African Centre of Excellence for Genomics of Infectious Diseases (Nigeria)	GISAID	EPI_ISL_1035827<br>EPI_ISL_1035826<br>EPI_ISL_1035825
COVID-19 Genomic Surveillance Regional Network (Latin America)	GISAID	EPI_ISL_2158821<br>EPI_ISL_2158802<br>EPI_ISL_2158810
COVID-19 Genomic Surveillance Regional Network (Latin America)	EMBL-EBI	ERR6279617<br>ERP130439<br>ERS6651658<br>ERX5914442
Rhode Island Department of Health/Broad Institute (SPHERES)	NCBI	SAMN18306978
Massachusetts General Hospital/Broad Institute (SPHERES)	NCBI	SAMN18309294
Flow Health/Broad Institute (SPHERES)	NCBI	SAMN18308763
New Brunswick Diagnostic Virology Reference Center/Public Health Agency of Canada (CanCOGeN)	NCBI	SAMN16784832
Toronto Invasive Bacterial Diseases Network/McMaster University (CanCOGeN)	NCBI	SAMN17505317
Bat coronavirus phylogeography- Université de La Réunion, UMR Processus Infectieux en Milieu Insulaire Tropical (PIMIT) and Field Museum of Natural History	NCBI	SAMN20400589<br>SAMN20400588

Conclusion

The collective response to the SARS-CoV-2 pandemic has resulted in an unprecedented deployment of genomic surveillance worldwide, bringing together public

health agencies, academic research institutions, and industry partners. This unified action provides opportunities to more effectively understand and respond to the pandemic. Yet it also provides an enormous challenge, as realizing the full potential of this opportunity will require standardisation and harmonization of data collection across these partners. With our SARS-CoV-2 metadata specification we have endeavored to create a mechanism for promoting consistent, standardised contextual data collection that can be applied broadly. We envision that given the increased uptake, this specification will improve the consistency of collected data, making information reusable by agencies as they continue working towards an increased understanding of SARS-CoV-2 epidemiology and biology, and harmonising them such that community-based data sharing efforts are not excessively burdened. We anticipate that the experience and lessons learned creating the specification package for SARS-CoV-2 will better enable the rapid development and deployment of pathogen-specific standards for public health pathogen genomic surveillance in the future.

Methods

The PHA4GE SARS-CoV-2 data specification was developed by first comparing existing metadata standards (e.g., MIxS, MIGS, the NIAID/BRC Sample Application Standard), various sequence repository submission requirements (e.g., GISAID, INSDC), as well as national and international case report forms.

A gap analysis was performed to identify SARS-CoV-2 public health surveillance data elements that were missing in available standards. Fields in existing standards that were deemed to be out of scope were excluded from the specification. Terms for pick lists were sourced from public health documents, the literature, and when available, various interoperable ontologies (OBO Foundry). The fields and terms from the gap analysis were structured in the collection template (.xlsx). Field definitions, guidance for use, examples and mappings to various standards were developed as part of a Reference Guide provided in a separate tab in the template workbook. Vocabulary lists were also provided in a separate tab in the template workbook to enable validation, and to enable users to add terms to pick lists as needed, according to instructions provided in the curation SOP. The specification was also encoded as a JavaScript Object Notation (JSON) file.

The specification was reviewed by public health, bioinformaticians and data standards experts from different public health agencies, research institutes and sequencing consortia and adapted according to feedback. Upon request by community members, versioned protocols for public repository submission were created and deposited in protocols.io.

The first version of the specification was made publicly available in August 2020 with a CC-BY 4.0 International attribution license. Iterative improvements were made to a development branch of the specification over the next 10 months as the pandemic evolved, and in response to user feedback and requests. The next major release (2.0) was made publicly available in May 2021. The PHA4GE template was incorporated into the contextual data harmonization, validation and transformation tool called The DataHarmonizer through a collaborative effort with the Hsiao Public Health Bioinformatics Laboratory (Simon Fraser University). Details regarding DataHarmonizer development can be found elsewhere (manuscript in preparation).

Availability and Requirements

The software used in this study is available on GitHub.
Project name: SARS-CoV-2-Contextual-Data-Specification
Project home page: https://github.com/pha4ge/SARS-CoV-2-Contextual-Data-Specification
Operating system: Platform independent
Programming language: Not applicable
Other requirements: Microsoft Excel
License: CC-BY 4.0 International
RRID: SCR_021378

List of abbreviations

ACEGID: African Center of Excellence for Genomics of Infectious Diseases; CanCOGeN: Canadian COVID Genomics Network; COG-UK: COVID-19 Genomics UK Consortium; COVID-19: coronavirus disease of 2019; EBI: European Bioinformatics Institute; EFO: Experimental Phenotype Ontology; EMBL-EBI: European Molecular Biology Laboratory's European Bioinformatics Institute; ENA: European Nucleotide Archive; FAIR: Findable Accessible Interoperable Reusable; GAZ: Gazetteer Ontology; GenEpiO: Genomic Epidemiology Ontology; GISAID: Global Initiative on Sharing All Influenza Data; HP: Human Phenotype Ontology; INSDC: International Nucleotide Sequence Database Collaboration; INSACOG: Indian SARS-CoV-2 Genomic Consortia; JSON: JavaScript Object Notation; LIMS: Laboratory Information Management System; MIGS: Minimum Information about a Genomic Sequence; MIxS: Minimum Information about any Sequence; MP: Mammalian Phenotype Ontology; NCBI: National Center for Biotechnology Information; NCBITaxon: NCBI Taxonomy Ontology; NCIT: National Cancer Institute Thesaurus; OBI: Ontology for Biological Investigations; OBO Foundry: Open Biological and Biomedical Ontology Foundry; PHA4GE: Public Health Alliance for

Genomic Epidemiology; SANBI: South African National Bioinformatics Institute; SARS-CoV-2: Severe Acute Respiratory Syndrome coronavirus 2; SOP: standard operating procedure; SPHERES: SARS-CoV-2 Sequencing for Public Health Emergency Response, Epidemiology and Surveillance; SRA: Sequence Read Archive; UBERON: Uber-Anatomy Ontology; UO: Unit Ontology

Declarations

Ethics approval and consent to participate

Not applicable.

Consent for publication

Not applicable.

Competing interests

The authors declare that they have no competing interests.

Funding

We wish to thank the Bill & Melinda Gates Foundation for supporting the establishment and work of the PHA4GE consortium. AJP and NFA gratefully acknowledge the support of the Biotechnology and Biological Sciences Research Council (BBSRC); and were supported by the Quadram Institute Bioscience BBSRC funded Core Capability Grant (project number BB/CCG1860/1) and the BBSRC Institute Strategic Programme Microbes in the Food Chain BB/R012504/1 and its constituent project BBS/E/F/000PR10352. FM was supported by a Donald Hill Family Fellowship in Computer Science. CIM was supported by the Fundação para a Ciência e Tecnologia (grant SFRH/BD/129483/2017). Work by EJG, RC, DD, and WWLH was funded by a Genome Canada Bioinformatics and Computational Biology 2017 Grant #286GET and a Genome Canada CanCOGeN grant E09CMA. The work of IKM, TB, and AJ was supported by the Intramural Research Program of the National Library of Medicine, National Institutes of Health.

Authors' contributions

EJG: Conceptualization, Methodology, Investigation, Software, Visualization, Writing - Original Draft Preparation, Validation, Supervision; RET: Methodology, Investigation, Software, Validation, Writing - Original Draft Preparation; CIM: Methodology, Software, Writing - Review & Editing; AJP: Methodology, Writing - Original Draft Preparation; NFA: Methodology, Software, Validation, Writing - Original Draft Preparation; DF: Methodology, Software; JC: Validation, Writing - Review & Editing; DP: Validation, Writing - Review & Editing; IDB: Validation, Writing - Review & Editing; DA: Software, Validation, Writing - Review & Editing; AC: Writing - Review & Editing; AGS: Software, Validation, Writing - Review & Editing; RC: Software, Validation; DD: Software, Validation; LSK: Validation, Writing - Review & Editing; AB: Methodology, Writing - Original Draft Preparation; IKM: Software, Validation, Writing - Review & Editing; TB: Software, Validation, Writing - Review & Editing; AJ: Software, Validation, Writing - Review & Editing; TRC: Validation, Writing - Review & Editing; SMN: Validation, Writing - Review & Editing; AAW: Writing - Review & Editing; PEO: Writing - Review & Editing; GHT: Writing - Review & Editing; SHT: Writing - Review & Editing; ARR: Writing - Review & Editing; BA: Writing - Review & Editing; DAM: Writing - Review & Editing; EH: Writing - Review & Editing; WWLH: Writing - Review & Editing; ATRV: Writing - Review & Editing; DRM: Conceptualization, Methodology, Visualization, Writing - Review & Editing, Funding Acquisition

Acknowledgements

The authors would like to thank the US Center for Disease Control and Prevention's Technical Outreach and Assistance for States Team (TOAST) for their feedback, support, and assistance in disseminating the PHA4GE specification package among US public health networks.

Figure Legends

Figure 1: Contextual data flow.

Contextual data can be captured and structured using the PHA4GE specification so that it can be more easily harmonized across different data sources and providers. Different subsets of the harmonized data can be 1) shared with public repositories e.g. GISAID and INSDC, 2) shared with trusted partners e.g. national sequencing consortia, public health partners, and 3) kept private and retained locally with the potential for sharing in the future for particular surveillance or research activities. While fields have been colour-coded in the template to indicate whether they are considered "required", "strongly recommended" and "optional", how the specification is implemented, and how,

if any, of the data is shared, is ultimately at the discretion of the user. Box 1 describes the information types covered in the full specification.

Figure 2: The PHA4GE specification is being implemented in CanCOGeN to harmonize contextual data across jurisdictions.
A) CanCOGeN is Canada's SARS-CoV-2 national genomic surveillance initiative. Canada has a decentralized health system, with one federal and 13 provincial/territorial public health jurisdictions. Provinces/Territories have authority over how data is collected, stored and shared. Every Canadian public health jurisdiction uses different collection instruments (e.g., case report forms), different data management systems, and different pipelines and software to perform bioinformatic analyses. Provinces/Territories share sequencing data and accompanying contextual data with the National Microbiology Lab's national SARS-CoV-2 genomics database (starred) according to a version of the PHA4GE specification for national surveillance activities. B) Excerpts from two different province-specific case collection forms. Sample type information is collected in data collection instruments using different fields, different terms, at different levels of granularity, using abbreviations and formats. C) An anonymised example of how the standard consistently structures contextual information and how it is being used for data sharing. The contextual data specification provides a wide variety of fields and pick lists of terms. In the example, the full set of standardised information shown would be shared by the province with the national database. Standardised information in bold would be shared with public repositories, however select data elements (underlined) would be withheld according to jurisdictional data sharing policies. The specification enables users to harmonise and integrate data provenance, sampling strategy criteria, epidemiological information, and methods.

Figure 3: Overview of how the PHA4GE SARS-CoV-2 contextual data specification can be integrated into public repository submission.
The PHA4GE collection template provides a one-stop shop for different data types that are important for global surveillance. The protocols provided as part of the specification package describe how PHA4GE fields can be mapped to different repository submission forms. Consensus sequences (FASTA), accompanied by a subset of PHAGE fields, can be submitted to the GISAID EpiCoV database (A). Consensus sequences (FASTA) (B) as well as raw/processed data (FASTQ, BAM) (C, D) can be submitted to INSDC databases (e.g., GenBank, SRA) with different subsets of PHA4GE fields as part of a BioSample record. BioSamples are propagated throughout INSDC databases.

References

[1] Coronavirus disease (COVID-19) – World Health Organization. https://www.who.int/emergencies/diseases/novel-coronavirus-2019. Accessed Jun. 21, 2021.

[2] Dong E, Du H, Gardner L. An interactive web-based dashboard to track COVID-19 in real time. *Lancet Infect. Dis.* 2020; 20: 533–534.

[3] The COVID-19 Genomics UK (COG-UK) consortium. An integrated national scale SARS-CoV-2 genomic surveillance network. Lancet Microbe. 2020;1:e99–e100.

[4] Cases, Data, and Surveillance. https://www.cdc.gov/coronavirus/2019-ncov/variants/spheres.html. Accessed Jun. 22, 2021.

[5] CanCOGeN (Genome Canada). https://www.genomecanada.ca/en/cancogen. Accessed Jun. 22, 2021.

[6] Laboratory Guidelines for the Detection and Diagnosis of COVID-19 Virus Infection - PAHO/WHO. https://www.paho.org/en/documents/laboratory-guidelines-detection-and-diagnosis-covid-19-virus-infection. Accessed Jun. 22, 2021.

[7] Candido DS et al. Evolution and epidemic spread of SARS-CoV-2 in Brazil. Science. *2020*;369:1255–1260.

[8] Zhao WM et al. The 2019 novel coronavirus resource. Yi Chuan Hered. 2020;42:212–221.

[9] NGS-SA: Network for Genomic Surveillance South Africa. http://www.krisp.org.za/ngs-sa/ngs-sa_network_for_genomic_surveillance_south_africa/. Accessed Jun. 22, 2021.

[10] AusTrakka. https://www.cdgn.org.au/austrakka. Accessed Jun. 22, 2021.

[11] Indian SARS-CoV-2 Genomic Consortia (INSACOG). http://dbtindia.gov.in/insacog. Accessed Jun. 21, 2021.

[12] Shu Y, McCauley J. GISAID: Global initiative on sharing all influenza data – from vision to reality. Eurosurveillance. 2017;22:30494.

[13] Karsch-Mizrachi I, Takagi T, Cochrane G, The International Nucleotide Sequence Database Collaboration. The international nucleotide sequence database collaboration. Nucleic Acids Res. 2018;46:D48–D51.

[14] Allard MW et al. Practical Value of Food Pathogen Traceability through Building a Whole-Genome Sequencing Network and Database. J. Clin. Microbiol. 2016;54:1975–1983.

[15] Kubota KA et al. PulseNet and the Changing Paradigm of Laboratory-Based Surveillance for Foodborne Diseases. Public Health Rep. 2019;134:22S-28S.

[16] Cook JA et al. Integrating Biodiversity Infrastructure into Pathogen Discovery and Mitigation of Emerging Infectious Diseases. Bioscience. 2020:70:531–534.

[17] Andersen KG, Rambaut A, Lipkin WI, Holmes EC, Garry RF. The proximal origin of SARS-CoV-2. Nat. Med. 2020;26:450–452.

[18] Gupta RK. Will SARS-CoV-2 variants of concern affect the promise of vaccines?. Nat. Rev. Immunol. 2021;21:340–341.

[19] Public Health England Technical Briefing 16: SARS-CoV-2 variants of concern and variants under investigation. https://assets.publishing.service.gov.uk/government/uploads/system/uploads/attachment_data/file/994839/Variants_of_Concern_VOC_Technical_Briefing_16.pdf. Accessed Jun. 22 2021.

[20] In silico evaluation of diagnostic assays: Los Alamos National Laboratory. https://covid19.edgebioinformatics.org/#/assayValidation. Accessed Jun. 22, 2021.

[21] Kuchinski KS. Mutations in emerging variant of concern lineages disrupt genomic sequencing of SARS-CoV-2 clinical specimens. medRxiv, 2021. doi: 10.1101/2021.06.01.21258181.

[22] Ganguli A et al. Rapid isothermal amplification and portable detection system for SARS-CoV-2. Proc. Natl. Acad. Sci. 2020;117:22727–22735.

[23] COVID-19 vaccine tracker and landscape. https://www.who.int/publications/m/item/draft-landscape-of-covid-19-candidate-vaccines. Accessed Jun. 22, 2021.
[24] Tillett RL et al. Genomic evidence for reinfection with SARS-CoV-2: a case study," Lancet Infect. Dis. 2021;21:52–58.
[25] Munnink BBO. Transmission of SARS-CoV-2 on mink farms between humans and mink and back to humans. Science. 2021;371:172–177.
[26] Lai C-C et al. COVID-19 in long-term care facilities: An upcoming threat that cannot be ignored," J. Microbiol. Immunol. Infect. 2020;53:444–446.
[27] D. Aggarwal *et al.*, "The role of genomics in understanding COVID-19 outbreaks in long term care facilities." OSF Preprints, Nov. 23, 2020. doi: 10.31219/osf.io/7y9rk.
[28] Murti M et al. Investigation of a severe SARS-CoV-2 outbreak in a long-term care home early in the pandemic. CMAJ. 2021;193:E681–E688.
[29] Dyal JW. COVID-19 Among Workers in Meat and Poultry Processing Facilities — 19 States, April 2020. MMWR Morb. Mortal. Wkly. Rep. 2020;69 doi: 10.15585/mmwr.mm6918e3.
[30] Günther T et al. EMBO Mol. Med. 2020;12 doi: 10.15252/emmm.202013296.
[31] Taylor J et al. Serial Testing for SARS-CoV-2 and Virus Whole Genome Sequencing Inform Infection Risk at Two Skilled Nursing Facilities with COVID-19 Outbreaks - Minnesota, April-June 2020. MMWR Morb. Mortal. Wkly. Rep. 2020;69:1288–1295.
[32] Loconsole D et al. Investigation of an outbreak of symptomatic SARS-CoV-2 VOC 202012/01-lineage B.1.1.7 infection in healthcare workers, Italy. Clin. Microbiol. Infect. Off. Publ. Eur. Soc. Clin. Microbiol. Infect. Dis., 2021. doi: 10.1016/j.cmi.2021.05.007.
[33] Frampton D et al. Genomic characteristics and clinical effect of the emergent SARS-CoV-2 B.1.1.7 lineage in London, UK: a whole-genome sequencing and hospital-based cohort study. Lancet Infect. Dis. 2021. doi: 10.1016/S1473-3099(21)00170-5.
[34] da Silva Filipe S et al. Genomic epidemiology reveals multiple introductions of SARS-CoV-2 from mainland Europe into Scotland. Nat. Microbiol. 2021;6:112–122.
[35] Munnink BBO et al. Rapid SARS-CoV-2 whole-genome sequencing and analysis for informed public health decision-making in the Netherlands. *Nat. Med.* 2020;26:1405–1410.
[36] du Plessis L et al. Establishment and lineage dynamics of the SARS-CoV-2 epidemic in the UK. Science. 2021;371:708–712.
[37] Githinji G et al. Tracking the introduction and spread of SARS-CoV-2 in coastal Kenya. medRxiv. 2020. doi: 10.1101/2020.10.05.20206730.
[38] Meredith LW et al. Rapid implementation of SARS-CoV-2 sequencing to investigate cases of health-care associated COVID-19: a prospective genomic surveillance study. Lancet Infect. Dis. 2020;20:1263–1271.
[39] Zhang W et al. Analysis of Genomic Characteristics and Transmission Routes of Patients With Confirmed SARS-CoV-2 in Southern California During the Early Stage of the US COVID-19 Pandemic. Jama Net. 2020;3:e2024191. doi: 10.1001/jamanetworkopen.2020.24191.
[40] Long S et al. Molecular Architecture of Early Dissemination and Massive Second Wave of the SARS-CoV-2 Virus in a Major Metropolitan Area. mBio. doi: 10.1128/mBio.02707-20.
[41] Geoghegan JL et al. Genomic epidemiology reveals transmission patterns and dynamics of SARS-CoV-2 in Aotearoa New Zealand. *Nat. Commun.* 2020;11:6351.
[42] Seemann T et al. Tracking the COVID-19 pandemic in Australia using genomics. Nat. Commun. 2020;11:4376.
[43] McLaughlin A et al. Early and ongoing importations of SARS-CoV-2 in Canada. medRxiv. 2021. doi: 10.1101/2021.04.09.21255131.
[44] Fauver JR et al Coast-to-Coast Spread of SARS-CoV-2 during the Early Epidemic in the United States. Cell. 2020;181:990-996.e5.
[45] Knock ES et al. Key epidemiological drivers and impact of interventions in the 2020

SARS-CoV-2 epidemic in England. Sci. Transl. Med. 2021. doi: 10.1126/scitranslmed.abg4262.
[46] C. R. Lane *et al.*, “Genomics-informed responses in the elimination of COVID-19 in Victoria, Australia: an observational, genomic epidemiological study,” *Lancet Public Health*, vol. 0, no. 0, Jul. 2021, doi: 10.1016/S2468-2667(21)00133-X.
[47] Issues with SARS-CoV-2 sequencing data - SARS-CoV-2 coronavirus / nCoV-2019 Genomic Epidemiology. Virological. https://virological.org/t/issues-with-sars-cov-2-sequencing-data/473. Accessed Jun. 22, 2021.
[48] Rayko M, Komissarov A. Quality control of low-frequency variants in SARS-CoV-2 genomes. bioRxiv. 2020. doi: 10.1101/2020.04.26.062422.
[49] Poon LLM, Leung CSW, Chan KH, Yuen KY, Guan Y, Peiris JSM. Recurrent mutations associated with isolation and passage of SARS coronavirus in cells from non-human primates. J. Med. Virol. 2005;76:435–440.
[50] Yilmaz P et al. Minimum information about a marker gene sequence (MIMARKS) and minimum information about any (x) sequence (MIxS) specifications. Nat. Biotechnol. 2011;29:415–420.
[51] Field D et al. The minimum information about a genome sequence (MIGS) specification. *Nat. Biotechnol.* 2008;26:541–547.
[52] Dugan VG et al. Standardized Metadata for Human Pathogen/Vector Genomic Sequences. PLOS ONE. 2014. doi: 10.1371/journal.pone.0099979.
[53] Smith B et al. The OBO Foundry: coordinated evolution of ontologies to support biomedical data integration. Nat. Biotechnol. 2007;vol. 25:1251–1255.
[54] Schriml LM et al. COVID-19 pandemic reveals the peril of ignoring metadata standards. Sci. Data. 2020;7:188.
[55] The PHA4GE SARS-CoV-2 Contextual Data Specification. https://github.com/pha4ge/SARS-CoV-2-Contextual-Data-Specification. Accessed Jun. 22, 2021.
[56] The OBO Foundry. http://www.obofoundry.org/ (accessed Jun. 22, 2021).
[57] PHA4GE - research group on protocols.io. Protocols.io. https://www.protocols.io/groups/pha4ge. Accessed Jun. 22, 2021.
[58] The DataHarmonizer. Hsiao Public Health Bioinformatics Lab. https://github.com/Public-Health-Bioinformatics/DataHarmonizer. Accessed Jun. 22, 2021.
[59] GET Africa – ONE AFRICA, ONE HEALTH, ONE DESTINY. https://www.getafrica.org/ Accessed Jun. 22, 2021.
[60] ACEGID Doctoral Research Fellows win PHA4GE Sub-Grant. https://acegid.org/2021/04/26/acegid-doctoral-research-fellows-win-pha4ge-sub-grant/ Accessed Jun. 22, 2021.
[61] Baobab LIMS. https://baobablims.org/. Accessed Jun. 22, 2021.
[62] SANBI – South African National Bioinformatics Institute. https://www.sanbi.ac.za/ Accessed Jun. 22, 2021.
[63] COVID-19 Genomic Surveillance Regional Network - PAHO/WHO. https://www.paho.org/en/topics/influenza/covid-19-genomic-surveillance-regional-network Accessed Jun. 22, 2021.
[64] Barrett T et al. BioProject and BioSample databases at NCBI: facilitating capture and organization of metadata. Nucleic Acids Res. 2012;40:D57-63.
[65] NCBI Pathogen Detection Portal. https://www.ncbi.nlm.nih.gov/pathogens/. Accessed Jun. 22, 2021.
[66] Compare Europe. https://www.compare-europe.eu/. Accessed Jun. 22, 2021.
[67] Dedicated SARS-CoV-2 BioSample submission package in the NCBI Submission Portal. https://ncbiinsights.ncbi.nlm.nih.gov/2021/05/11/sars-cov-2-biosample-submission-package/. Accessed Jun. 22, 2021.

Figure 1

Click here to access/download;Figure;Figure 1_spec content.docx

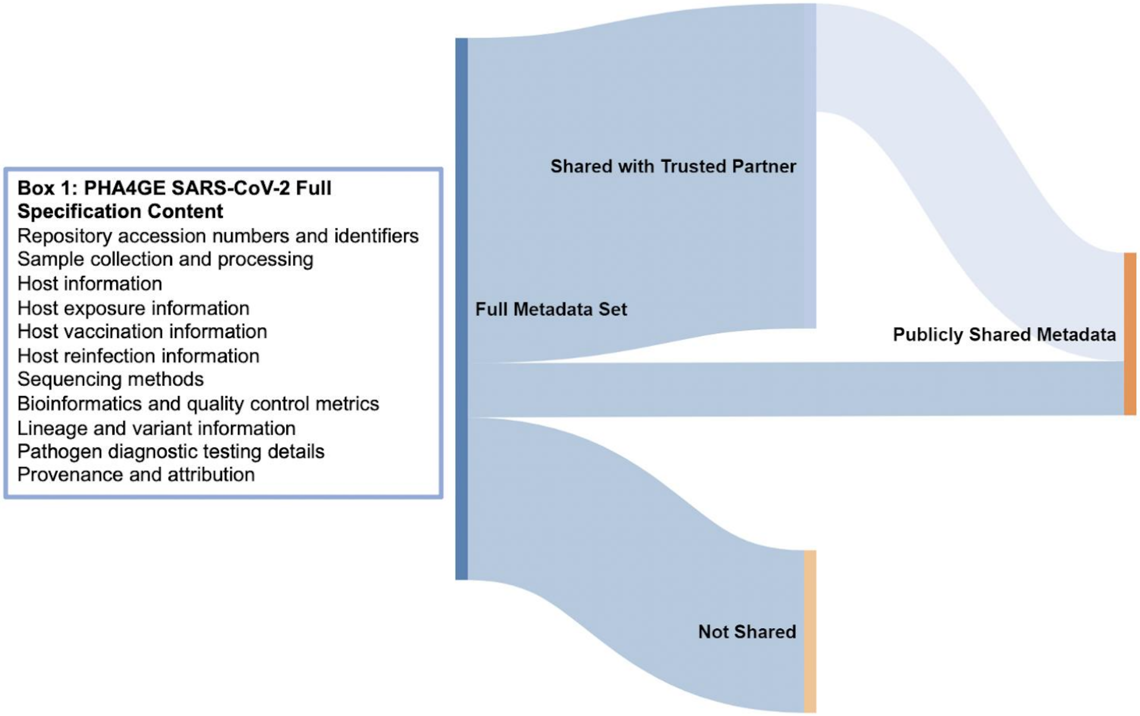

Figure 2

Click here to access/download;Figure;Figure 2_worked example.docx

A

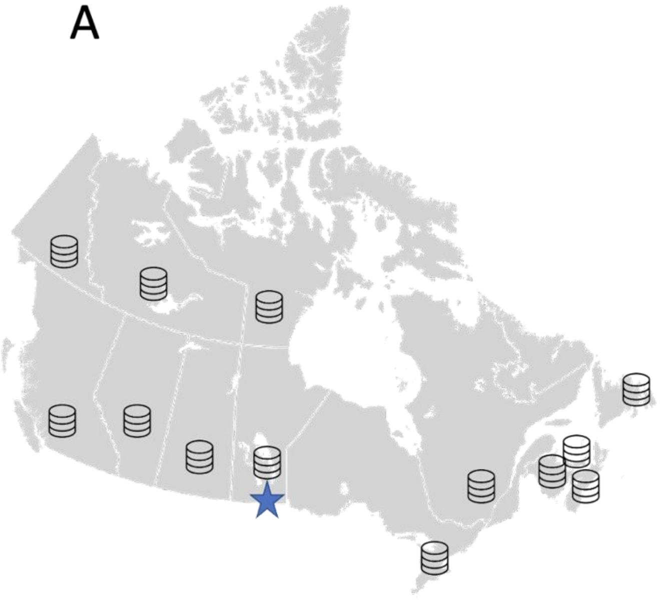

B

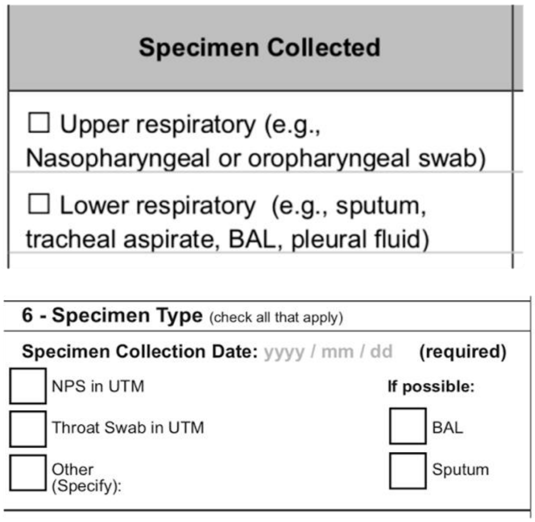

C

Anonymised Example Data:

Province A screens travelers as part of an international border testing program. A sample is selected by the lab team (Johnny Bloggs, Bhav Singh, Tina Lee) for sequencing as part of the **travel surveillance** program. The sample, collected **on November 13 2020**, is a **nasal swab** from an individual who **shares a household with a known case** that **recently traveled to country X**. The individual was **asymptomatic**. Diagnostic RT-qPCR testing based on the E gene yielded a **CT value of 23**. The individual was a **43 year old female**. The lab implements the **ARTIC protocol** to perform amplicon sequencing on an **Illumina MiSeq** using the **primer scheme described by Freed et al (2020), and ncov-tools** for bioinformatic processing and analysis.

Standardized Contextual Data:

specimen collector sample ID: provA_12345
sample collected by: Province A Public Health Lab
sample collector contact email: provlabA@lab.ca
sequence submitted by: Province A Public Health Lab
sequence submitter contact email: provlabA@lab.ca
geo_loc_name (country): Canada
geo_loc_name (state/province/region): Province A
sample collection date: 2020-11-13
anatomical site: Nasopharynx (NP)
collection device: Swab
purpose of sampling: Diagnostic testing
purpose of sequencing: Travel-associated surveillance
host (scientific name): Severe acute respiratory syndrome coronavirus 2
host disease: COVID-19
host age: 43
host age bin: 40 - 49
host age unit: year
host gender: Female
host health state: Asymptomatic
exposure setting: Contact with known COVID-19 case; Contact with person that travelled
sequencing instrument: Illumina MiSeq
sequencing protocol name: ARTIC protocol
amplicon pcr primer scheme: Freed at al (2020)
amplicon size: 1200 bp
raw sequence data processing method: https://github.com/jts/ncov-tools
dehosting method: https://github.com/jts/ncov-tools
consensus sequence software name: Freebayes
consensus sequence software version: 1.3.2
gene name 1: E gene (orf4)
diagnostic pcr Ct value 1: 23
authors: Johnny Bloggs, Bhav Singh, Tina Lee

Figure 3

Click here to access/download;Figure;Figure 3_integration.docx

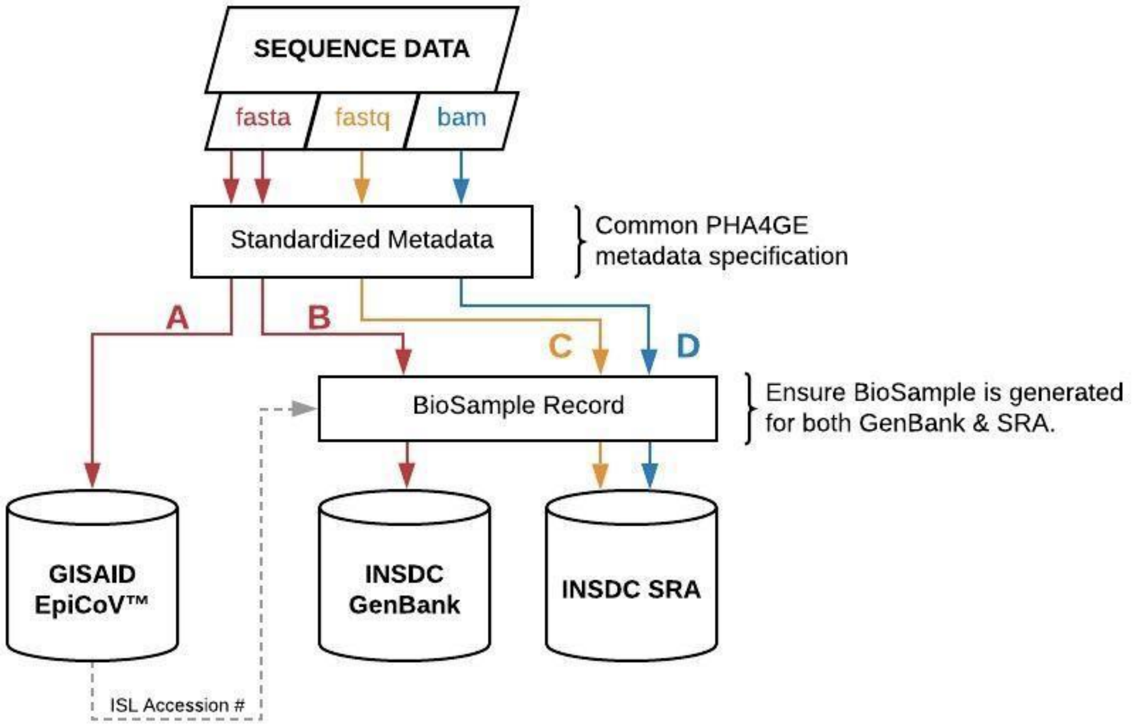

Supplement: giac003_GIGA-D-21-00246_Original_Submission [file giac003_giga-d-21-00246_original_submission.pdf]
