# Supplementary material for: Future-proofing and maximizing the utility of metadata: The PHA4GE SARS-CoV-2 contextual data specification package
Source: Gigascience. 2022 Feb 16;11:giac003. doi: 10.1093/gigascience/giac003 (PMC8847733; doi:10.1093/gigascience/giac003)
Supplement: giac003_Response_to_Reviewer_Comments_Original_Submission [file giac003_response_to_reviewer_comments_original_submission.pdf]

Author's Response To Reviewer Comments

Close

Re: GIGA-D-21-00246
Future-proofing and maximizing the utility of metadata: The PHA4GE SARS-CoV-2 contextual data specification package
Emma Griffiths; Ruth E Timme; Catarina Inês Mendes; Andrew J Page; Nabil-Fareed Alikhan; Dan Fornika; Finlay Maguire; Josefina Campos; Daniel Park; Idowu B Olawoye; Paul E Oluniyi; Dominique Anderson; Alan Christoffels; Anders Gonçalves da Silva; Rhiannon Cameron; Damion Dooley; Lee S Katz; Allison Black; Ilene Karsch-Mizrachi; Tanya Barrett; Anjanette Johnston; Thomas R Connor; Samuel M Nicholls; Adam A Witney; Gregory H Tyson; Simon H Tausch; Amogelang R Raphenya; Brian Alcock; David M Aanensen; Emma Hodcroft; William WL Hsiao; Ana Tereza R Vasconcelos; Duncan R MacCannell

Dear Editor and Reviewers,

Thank you for suggestions and feedback. We took it all very much to heart and have made major updates based on Reviewer #3’s suggestions (see v 3.0 on GitHub) which we believe have greatly improved the specification, and hopefully have addressed all other issues. On that note, we had previously submitted the resource to SciCrunch.org, and have now also submitted the resource to bio.tools.
Please see our point-by-point responses to reviewer comments below.

Reviewer reports:
Reviewer #1: SARS-CoV-2 sequencing, analysis, and open sharing have played a crucial role in a number of developments during the pandemic. The authors have identified a need for a fit-for-purpose, open-source SARS-CoV-2 contextual data specification. They incorporates existing community standards with an emphasis on SARS-CoV-2 public health needs and ensuring privacy while maximizing information content and interoperability across datasets and databases to better enable analyses to fight COVID-19. The research is very important both for theoretical studies and clinical therapy of COVID-19. To facilitate timely access of research community to this package, I strongly recommend its publication as it is.

PHA4GE Response: Thank you! We greatly appreciate your support of our work and advocacy for its importance.

Reviewer #2: The work described in the manuscript - a specification of metadata to be provided for SARS-CoV-2 sequencing data - is of great relevance for open data science in the ongoing pandemic and beyond. Harmonization of metadata, as encouraged by the submitted work, lays the foundation for proper data analysis and interpretation across pathogen genome sequencing initiatives, not just in the case of SARS-CoV-2 but in general.
As stated by the authors, adoption of their specification by data providers cannot be enforced, but the authors have done a remarkable job at making it easy to adhere to it for anybody interested: their specification template in the forms of an xlsx spreadsheet and a DataHarmonizer template, the provided list of specification-compliant records in public databases, and their efforts to collaborate with public data repositories such as INSDC members are very valuable efforts in this direction.
By hosting the specification on a collaborative, version-control platform, the authors are also providing the opportunity, for e.g. data analysts, to suggest improvements. Because of this possibility, I feel that publication of the manuscript should not be delayed by arguing about individual fields of the specification or the exact wording of the accompanying help text, which can be handled much more efficiently through issues and pull requests against the public repository.
Hence, I support the publication of the manuscript after the following truely minor comments have been addressed:

- in the section "Availability and requirements / Other requirements"
the product name Microsoft Excel should be replaced with "xlsx-compatible spreadsheet software",

or a similar general term.

PHA4GE Response: In the "Availability and requirements / Other requirements", we have replaced "Microsoft Excel" with "xlsx-compatible spreadsheet software" exactly as suggested.

- in the legend of Figure 1, "and how, if any, of the data" should be corrected to "and which parts, if any, of the data"

PHA4GE Response: In the figure legend of Figure 1, we have replaced "and how, if any, of the data" with "and which parts, if any, of the data" as suggested.

- in the legend of Figure 3, PHA4GE is misspelled once as "PHAGE"

PHA4GE Response: We have corrected the misspelling of PHA4GE in Figure 3.

Reviewer #3: Overall the manuscript it well written and provides a valuable resource to the community, it is clear that the checklist has been given a great deal of thought and that it has been tested in real-world situations with the iterative changes made resulting in a set of recommendations that could lead to greater interoperability of these very important data.

While I do have quite a number of minor concerns that I believe would improve the manuscript and PHA4GE Excel template, there is nothing that should prevent the acceptance of this manuscript. Here I list the minor points that I would like to see addressed:

1 - In table 1, there are links to a number of protocols.io URLs, these should be updated to use the DOIs instead.

PHA4GE Response: We have replaced the protocol URLs with the DOIs as suggested.

2 - Table 2 has the title "Minimal (required) contextual data fields", but some of the fields listed in table 2 are in the template spreadsheet as recommended (not required), e.g. purpose of sequencing, purpose of sequencing details. Please check which is correct and amend as appropriate.

PHA4GE Response: We have corrected the "Minimal (required) contextual data fields" table. There are now 14 required fields, and "purpose of sequencing details" has been removed. Corresponding updates were made in the revised collection template.

3 - In table 3, the 3rd row "COVID-19 Genomic Surveillance Regional Network (Latin America) EMBL-EBI ERR6279617, ERP130439, ERS6651658, ERX5914442" contains details of an ENA submitted example. I am unsure why all 4 accessions for the various part of the same submission (run, experiment, sample and project level metadata) are given? I believe the only relevant accession here is that of the sample metadata, and in that case the BioSample accession () should be quoted instead of the multiple ENA accessions.

PHA4GE Response: Thank you for spotting this! We have replaced all of the previous accessions with the BioSample accession "SAMEA8968916".

The remainder of my concerns are related to the Excel file "PHA4GE SARS-CoV-2 Contextual Data Template.xlsx":

4 - With the stated intention of the specification being to provide a mechanism for consistent metadata to aid integration of data, it would be of a great benefit for the "pick lists" given to make better use of ontologies/vocabularies. Clearly some of the selected values in those pick-lists are from well curated sources, but no links have been included which will mean someone will have to re-do those mappings again in the future. I strongly recommend that where possible the CURIE(https://www.w3.org/TR/curie/) of the value be included, e.g. Blood [UBERON:0000178]
Where a suggested "pick list" value has not been selected from (and referenced to) a curated source the authors should include adequate descriptions of the suggested term to avoid any unnecessary confusion about the meanings. For example in "purpose of sampling"; how does "Cluster/Outbreak Investigation" differ from either "Research" or "Surveillance"? Having definitions on the picklist terms will allow users to pick the most appropriate value(s).

PHA4GE Response: This was an excellent suggestion and we have done a major update and new release based on these suggestions. We have done a lot of work to revise the pick lists and the reference guide to include ontology identifiers corresponding to the terms. We have mapped all fields and terms to existing ontologies and included those identifiers in the field-level reference guide as well as a newly created term-level reference guide so that all of the fields and terms have definitions. Where terms could not be mapped to existing ontologies, we have worked with ontology developers to create new terms and have included those new identifiers where appropriate. Every pick list term is now in the suggested format e.g. Blood [UBERON:0000178]. All of these updates can be found in version 3.0 of the package on GitHub. We have included the new pick list format in the examples in the reference guide as well as in the worked example in Figure 2 on the manuscript. We continue to collaborate with COVID-related ontology developers to build additional axioms and cross references between these ontologies.

5 - The null value options appear to be from the INSDC suggested null values, which is a fine choice, but they should be defined here or reference the INSDC as the source of meanings of those.

PHA4GE Response: The INSDC null values have been ontologized and definitions are included in the term-level reference guide.

6 - In some cases the null value options include "missing" and "unknown", please clarify the difference? the use of the controlled vocabulary for null terms is to avoid this sort of confusion, so introducing it again in the "pick lists" should be avoided is possible.

PHA4GE Response: We re-evaluated the null values are decided to remove “unknown”. This null value is very often used in public health, but since “Missing” could be used to replace it without much change in meaning/interpretation, we decided to remove it.

7 - I have concerns over the usage and definitions of these 3 related terms in the checklist:
anatomical material
anatomical part
body product

The pick list values for "anatomical material" are all either anatomical parts OR body products, and the definitions do not clarify the differences.
There are only 2 distinct terms in the GSC-MIxS human-associated checklist that I think are equivalents to the anatomical part and body product terms listed in this package, perhaps "anatomical material" is not required?:
host body site - Name of body site where the sample was obtained from, such as a specific organ or tissue (tongue, lung etc...). For foundational model of anatomy ontology (fma) (v 4.11.0) or Uber-anatomy ontology (UBERON) (v releases/2014-06-15) terms, please see http://purl.bioontology.org/ontology/FMA or http://purl.bioontology.org/ontology/UBERON
host body product - Substance produced by the body, e.g. Stool, mucus, where the sample was obtained from. For foundational model of anatomy ontology (fma) or Uber-anatomy ontology (UBERON) terms, please see https://www.ebi.ac.uk/ols/ontologies/fma or https://www.ebi.ac.uk/ols/ontologies/uberon

PHA4GE Response: Thank you for bringing up these valuable points.
In the specification, anatomical part is defined as “An anatomical part of an organism e.g. oropharynx.”, while anatomical material is defined as “A substance obtained from an anatomical part of an organism e.g. tissue, blood.”. These fields are distinguished by the part specifying the named anatomical structure of the body/organism, while the material describes what of that anatomical structure was taken/removed/sampled which could be the entire structure, the contents of an organ, fluid (specific to the structure or a mixture depending on the collection method), etc. The part specifies the “where” in the organism, while the material specifies the “what”. These different dimensions are critical as the same anatomical structure can be sampled in different ways which can bias results, and therefore warrant separate fields. E.g. anatomical part: cecum, anatomical material: tissue vs anatomical part: cecum, anatomical material: organ contents. Body products in the specification are defined as “ A substance excreted/secreted from an organism e.g. feces, urine, sweat.”. These are substances produced by the body and only transiently present in the body/organism as opposed to anatomical structures which are more permanent. These fields and differentiae are part of an ISO standard undergoing final stages of international review, and also form part of the NCBI BioSample for SARS-CoV-2 (we believe Lynn Schriml is including these in an upcoming updated MIxS SARS-CoV-2 standard).

Having carefully considered the reviewer’s thoughtful points, we still felt the definitions, in combination with the examples in the reference guide and curation SOP, adequately differentiated these fields and so felt it appropriate not to make any alterations to these fields.

8 - With regards to the Reference guide tab in the spreadsheet, the authors have include a column for mapping to "MIxS v5" and another for mapping to "MIGS Virus, Host-Associated Field". I applaud the efforts to provide such mappings, thank you. However, the GSC MIGS virus checklist is in fact a part of the MIxS family of checklists, so there should be no differences between those columns. In the attached spreadsheet I have suggested that those two columns be replaced by 1 column called "GSC MIGS Virus, Human-associated (v5)", in addition I have added in more mappings that appear to have been missed.

PHA4GE Response: Thank you for these corrections. We have removed the MIxS mappings, and only included the MIGS column. We have chosen to include the “MIGS Virus, Host-Associated Field” package over the Human-associated package as the specification applies to all hosts not just humans (e.g. bats, pangolins, food-production animals etc. We include a BioSample for a bat phylogeny dataset in our list of example implementations, for example), and so we felt the Host-associated package was most appropriate. We hope we have provided the correct mappings to this package, using the mappings you have indicated.

9 - Finally, there are a number of other more minor points to do with the Excel template/reference guide, I have added many comments in the relevant cells of the spreadsheet (and highlighted those cells in red), some are just comments for your information about the GSC checklists, others are things that you may be able to address.

PHA4GE Response to template-related comments:
Host specimen voucher: We DO expect host specimens if the hosts are animals. In fact, one of the groups implementing the specification is examining coronaviruses in museum bat collections, therefore we need to be able to specify/distinguish these identifiers.
Sample collection date: Since we are using ontology-based definitions, the rule is not to specify formats in the definitions(which are meant to be universal and not particular to any specification) but rather specify those as guidance in whatever spec is using those definitions. That is the convention we have followed in the specification and so have not included the YYYY-MM-DD recommendation in the definition.
Comments regarding granularity and relation to INSDC submission: Thank you for all the additional mappings and insights. I hope we have represented the MIGS standard appropriately. These mappings were meant in no way as a criticism, only to highlight that our standards are meant to do different things. We recognize that the MIxS/MIGS packages were meant to provide standards for INSDC submissions and so, for example, may not track identifiers and granularity of geographic locations in the way we have done in the PHA4GE specification. This is one of the concepts that we are trying to draw out. It is important for public health agencies to track samples that move between labs, information that moves between databases, and hosts that become infected in different scenarios, for epidemiological analysis, auditability, and for tracking chain of custody. Information may need to be tracked internally that would not be shared with public repositories, and so we have attempted to fill gaps in standards identified through a public health lens by providing additional data structures compatible with standards for public sharing.

Close
