# Supplementary material for: Future-proofing and maximizing the utility of metadata: The PHA4GE SARS-CoV-2 contextual data specification package
Source: Gigascience. 2022 Feb 16;11:giac003. doi: 10.1093/gigascience/giac003 (PMC8847733; doi:10.1093/gigascience/giac003)
Supplement: giac003_Reviewer_2_Report_Original_Submission — Wolfgang Maier -- 9/2/2021 Reviewed [file giac003_reviewer_2_report_original_submission.pdf]

Reviewer Report

Title: Future-proofing and maximizing the utility of metadata: The PHA4GE SARS-CoV-2 contextual data specification package

Version: Original Submission **Date:** 9/2/2021

Reviewer name: Wolfgang Maier

Reviewer Comments to Author:

The work described in the manuscript - a specification of metadata to be provided for SARS-CoV-2 sequencing data - is of great relevance for open data science in the ongoing pandemic and beyond. Harmonization of metadata, as encouraged by the submitted work, lays the foundation for proper data analysis and interpretation across pathogen genome sequencing initiatives, not just in the case of SARS-CoV-2 but in general.
As stated by the authors, adoption of their specification by data providers cannot be enforced, but the authors have done a remarkable job at making it easy to adhere to it for anybody interested: their specification template in the forms of an xlsx spreadsheet and a DataHarmonizer template, the provided list of specification-compliant records in public databases, and their efforts to collaborate with public data repositories such as INSDC members are very valuable efforts in this direction.
By hosting the specification on a collaborative, version-control platform, the authors are also providing the opportunity, for e.g. data analysts, to suggest improvements. Because of this possibility, I feel that publication of the manuscript should not be delayed by arguing about individual fields of the specification or the exact wording of the accompanying help text, which can be handled much more efficiently through issues and pull requests against the public repository.
Hence, I support the publication of the manuscript after the following truely minor comments have been addressed:
- in the section "Availability and requirements / Other requirements"
the product name Microsoft Excel should be replaced with "xlsx-compatible spreadsheet software",
or a similar general term.
- in the legend of Figure 1, "and how, if any, of the data" should be corrected to "and which parts, if any, of the data"
- in the legend of Figure 3, PHA4GE is misspelled once as "PHAGE"

Level of Interest

Please indicate how interesting you found the manuscript: Choose an item.

Quality of Written English

Please indicate the quality of language in the manuscript: Choose an item.

Declaration of Competing Interests

Please complete a declaration of competing interests, considering the following questions:

- Have you in the past five years received reimbursements, fees, funding, or salary from an organisation that may in any way gain or lose financially from the publication of this manuscript, either now or in the future?
- Do you hold any stocks or shares in an organisation that may in any way gain or lose financially from the publication of this manuscript, either now or in the future?
- Do you hold or are you currently applying for any patents relating to the content of the manuscript?
- Have you received reimbursements, fees, funding, or salary from an organization that holds or has applied for patents relating to the content of the manuscript?
- Do you have any other financial competing interests?
- Do you have any non-financial competing interests in relation to this paper?

If you can answer no to all of the above, write 'I declare that I have no competing interests' below. If your reply is yes to any, please give details below.

I declare that I have no competing interests

I agree to the open peer review policy of the journal. I understand that my name will be included on my report to the authors and, if the manuscript is accepted for publication, my named report including any attachments I upload will be posted on the website along with the authors' responses. I agree for my report to be made available under an Open Access Creative Commons CC-BY license (http://creativecommons.org/licenses/by/4.0/). I understand that any comments which I do not wish to be included in my named report can be included as confidential comments to the editors, which will not be published.

Choose an item.

To further support our reviewers, we have joined with Publons, where you can gain additional credit to further highlight your hard work (see: https://publons.com/journal/530/gigascience). On publication of this paper, your review will be automatically added to Publons, you can then choose whether or not to claim your Publons credit. I understand this statement.

Yes Choose an item.
