# Supplementary material for: Future-proofing and maximizing the utility of metadata: The PHA4GE SARS-CoV-2 contextual data specification package
Source: Gigascience. 2022 Feb 16;11:giac003. doi: 10.1093/gigascience/giac003 (PMC8847733; doi:10.1093/gigascience/giac003)
Supplement: giac003_Reviewer_3_Report_Original_Submission — Christopher Hunter, Ph.D. -- 9/9/2021 Reviewed [file giac003_reviewer_3_report_original_submission.pdf]

Reviewer Report

Title: Future-proofing and maximizing the utility of metadata: The PHA4GE SARS-CoV-2 contextual data specification package

Version: Original Submission **Date:** 9/9/2021

Reviewer name: Christopher Hunter, Ph.D.

Reviewer Comments to Author:

Overall the manuscript it well written and provides a valuable resource to the community, it is clear that the checklist has been given a great deal of thought and that it has been tested in real-world situations with the iterative changes made resulting in a set of recommendations that could lead to greater interoperability of these very important data.

While I do have quite a number of minor concerns that I believe would improve the manuscript and PHA4GE Excel template, there is nothing that should prevent the acceptance of this manuscript. Here I list the minor points that I would like to see addressed:

1 - In table 1, there are links to a number of protocols.io URLs, these should be updated to use the DOIs instead.

2 - Table 2 has the title "Minimal (required) contextual data fields", but some of the fields listed in table 2 are in the template spreadsheet as recommended (not required), e.g. purpose of sequencing, purpose of sequencing details. Please check which is correct and amend as appropriate.

3 - In table 3, the 3rd row "COVID-19 Genomic Surveillance Regional Network (Latin America) EMBL-EBI ERR6279617, ERP130439, ERS6651658, ERX5914442" contains details of an ENA submitted example. I am unsure why all 4 accessions for the various part of the same submission (run, experiment, sample and project level metadata) are given? I believe the only relevant accession here is that of the sample metadata, and in that case the BioSample accession (SAMEA8968916) should be quoted instead of the multiple ENA accessions.

The remainder of my concerns are related to the Excel file "PHA4GE SARS-CoV-2 Contextual Data Template.xlsx":

4 - With the stated intention of the specification being to provide a mechanism for consistent metadata to aid integration of data, it would be of a great benefit for the "pick lists" given to make better use of ontologies/vocabularies. Clearly some of the selected values in those pick-lists are from well curated sources, but no links have been included which will mean someone will have to re-do those mappings again in the future. I strongly recommend that where possible the CURIE(https://www.w3.org/TR/curie/) of the value be included, e.g. Blood [UBERON:0000178]

Where a suggested "pick list" value has not been selected from (and referenced to) a curated source the authors should include adequate descriptions of the suggested term to avoid any unnecessary confusion about the meanings. For example in "purpose of sampling"; how does "Cluster/Outbreak Investigation" differ from either "Research" or "Surveillance"? Having definitions on the picklist terms will allow users to pick the most appropriate value(s).

5 - The null value options appear to be from the INSDC suggested null values, which is a fine choice, but

they should be defined here or reference the INSDC as the source of meanings of those.
6 - In some cases the null value options include "missing" and "unknown", please clarify the difference? the use of the controlled vocabulary for null terms is to avoid this sort of confusion, so introducing it again in the "pick lists" should be avoided is possible.
7 - I have concerns over the usage and definitions of these 3 related terms in the checklist:
anatomical material
anatomical part
body product
The pick list values for "anatomical material" are all either anatomical parts OR body products, and the definitions do not clarify the differences.
There are only 2 distinct terms in the GSC-MIxS human-associated checklist that I think are equivalents to the anatomical part and body product terms listed in this package, perhaps "anatomical material" is not required?:
host body site - Name of body site where the sample was obtained from, such as a specific organ or tissue (tongue, lung etc...). For foundational model of anatomy ontology (fma) (v 4.11.0) or Uber-anatomy ontology (UBERON) (v releases/2014-06-15) terms, please see http://purl.bioontology.org/ontology/FMA or http://purl.bioontology.org/ontology/UBERON
host body product - Substance produced by the body, e.g. Stool, mucus, where the sample was obtained from. For foundational model of anatomy ontology (fma) or Uber-anatomy ontology (UBERON) terms, please see https://www.ebi.ac.uk/ols/ontologies/fma or https://www.ebi.ac.uk/ols/ontologies/uberon
8 - With regards to the Reference guide tab in the spreadsheet, the authors have include a column for mapping to "MIxS v5" and another for mapping to "MIGS Virus, Host-Associated Field". I applaud the efforts to provide such mappings, thank you. However, the GSC MIGS virus checklist is in fact a part of the MIxS family of checklists, so there should be no differences between those columns. In the attached spreadsheet I have suggested that those two columns be replaced by 1 column called "GSC MIGS Virus, Human-associated (v5)", in addition I have added in more mappings that appear to have been missed.
9 - Finally, there are a number of other more minor points to do with the Excel template/reference guide, I have added many comments in the relevant cells of the spreadsheet (and highlighted those cells in red), some are just comments for your information about the GSC checklists, others are things that you may be able to address.

Level of Interest

Please indicate how interesting you found the manuscript: Choose an item.

Quality of Written English

Please indicate the quality of language in the manuscript: Choose an item.

Declaration of Competing Interests

Please complete a declaration of competing interests, considering the following questions:

- Have you in the past five years received reimbursements, fees, funding, or salary from an organisation that may in any way gain or lose financially from the publication of this manuscript, either now or in the future?
- Do you hold any stocks or shares in an organisation that may in any way gain or lose financially from the publication of this manuscript, either now or in the future?
- Do you hold or are you currently applying for any patents relating to the content of the manuscript?
- Have you received reimbursements, fees, funding, or salary from an organization that holds or has applied for patents relating to the content of the manuscript?
- Do you have any other financial competing interests?
- Do you have any non-financial competing interests in relation to this paper?

If you can answer no to all of the above, write 'I declare that I have no competing interests' below. If your reply is yes to any, please give details below.

I declare that I am an employee of GigaScience Press, and on the board of the Genomics Standards Consortium. I received no compensation (financial or otherwise) for performing this peer review. My review is impartial and unbiased.

I agree to the open peer review policy of the journal. I understand that my name will be included on my report to the authors and, if the manuscript is accepted for publication, my named report including any attachments I upload will be posted on the website along with the authors' responses. I agree for my report to be made available under an Open Access Creative Commons CC-BY license (http://creativecommons.org/licenses/by/4.0/). I understand that any comments which I do not wish to be included in my named report can be included as confidential comments to the editors, which will not be published.

Choose an item.

To further support our reviewers, we have joined with Publons, where you can gain additional credit to further highlight your hard work (see: https://publons.com/journal/530/gigascience). On publication of this paper, your review will be automatically added to Publons, you can then choose whether or not to claim your Publons credit. I understand this statement.

Yes Choose an item.
